# Efficient, complete biosynthesis of isoflavonoid calycosin-7-glucoside by metabolically engineered yeast cell factories

**DOI:** 10.3389/fbioe.2026.1875470

**Published:** 2026-07-09

**Authors:** Weifeng Zhang, Mingyuan Xu, Linlin Wang, Xue Qiao, Song Yang, Yi Liu, Quanli Liu, Xuefeng Lu

**Affiliations:** 1 School of Life Sciences, Qingdao Agricultural University, Qingdao, China; 2 State Key Laboratory of Photoelectric Conversion and Utilization of Solar Energy, Qingdao Institute of Bioenergy and Bioprocess Technology, Chinese Academy of Sciences, Qingdao, China; 3 Shandong Energy Institute, Qingdao, China; 4 University of Chinese Academy of Sciences, Beijing, China; 5 State Key Laboratory of Natural and Biomimetic Drugs, School of Pharmaceutical Sciences, Peking University, Beijing, China

**Keywords:** calycosin-7-glucoside, carbon source, cofactor, directed evolution, isoflavonoid, metabolic engineering, *Saccharomyces cerevisiae*

## Abstract

Plant isoflavonoids constitute a class of secondary metabolites that hold great potential in nutraceutical and pharmaceutical industries, considering their prominent human health-promoting properties. As a result, there is increasing interest in establishing alternative sources to these bioactive molecules to cope with the uneconomic and non-sustainable nature of traditional crop-based production. Here, we present the construction of a yeast platform that can *de novo* produce isoflavonoid calycosin-7-glucoside (CG), one of the quality markers for important medicinal *Astragalus* herbs. Through identifying active plant catalytic enzymes, optimizing carbon sources, utilizing and providing key precursor daidzein, and engineering biosynthesis of key metabolic cofactors, production of 47.60 mg/L CG from simple carbon sources was achieved in shake flask fermentations. In addition, we showcased the feasibility of modifying the substrate preference of selected plant glycosyltransferase by rational protein engineering. Our work provides an important step toward developing industrial-scale biomanufacturing of CG and may be extended to engineer complete biosynthesis of other value-added isoflavonoids in microbes.

## 1 Introduction

Plant isoflavonoids represent a large class of secondary metabolites that contain two phenyl rings linked together with a 3-carbonated heterocyclic ring and one of the phenyl rings attached to the three-position of the heterocyclic structure (Steele et al., 1999; Wang, 2011). These compounds exhibit various human health-promoting properties and are widely used as food ingredients and pharmaceuticals (Sharma and Ramawat, 2013). Puerarin, for example, is an active natural product extracted from the traditional Chinese medicine *Pueraria lobata* and has been applied in the treatment of coronary heart disease (Jiang et al., 2022). The bioactive isoflavonoid glycitein, which was found in *Glycine max*, has the potential for treating Parkinson’s disease (Zhao et al., 2025). However, present access to isoflavonoids is limited as a result of low natural abundance and uneven distribution of plant cultivations; thus, it is important to expand their sources to meet the constantly increasing market demand.

In contrast to traditional plant extraction and chemical synthesis, which are either inefficient or environmentally unfriendly, microbial biosynthesis is a promising route to obtain plant natural products in more sustainable and economical ways. Recently, significant production of different isoflavonoid molecules, including puerarin (Liu et al., 2021), biochanin A (Tan et al., 2026), glyceollin (Sun et al., 2025), 3′-hydroxygenstein (Tan et al., 2025), and medicarpin (Lu et al., 2023), was successfully demonstrated by grafting known plant biosynthetic pathways into well-characterized chassis cell *Saccharomyces cerevisiae*, together with performance improvement of resultant strains. While the realized low titers require more engineering efforts to enable industrial-scale bioproduction, these microbial cell factories unquestionably provide the foundation for further developing microbial biosynthesis of isoflavonoids with more complex structures.

In plants, isoflavonoid biosynthesis is generally classified as secondary metabolism, which is characterized by relatively low catalytic activities of involved enzymes and obvious inducibility in response to external stimulus (Ferrer et al., 2008; Du et al., 2010; Wang, 2011). In other words, endogenous metabolism of selected microbial chassis and performance of downstream plant enzymes need to be optimized in parallel to enable efficient heterologous production of these chemicals. Previously, many achievements have been made with respect to high-level production of plant natural products. For example, combining increased supply of key primary metabolite erythrose 4-phosphate of *S. cerevisiae* with enhanced expression of foreign functional genes, production of *para*-coumaric acid, a platform chemical for plant flavonoid biosynthesis, was improved remarkably to the gram scale (Liu et al., 2019). Moreover, *de novo* biosynthesis of bisbenzylisoquinoline alkaloids (BisBIAs) has been achieved in yeast by systematic metabolic engineering, including enhancing precursor synthesis and introducing a heterologous downstream catalytic element (Payne et al., -2021). To address the issue of low BisBIAs production, the rate-limiting steps were identified and resolved by increasing the copy number of key genes and protein engineering. The BisBIA titer increased by almost 10,000-fold together with culture condition optimization (Payne et al., 2021). Considering that plant natural products share a highly conserved biochemical logic, that is, their amazing structural and functional diversity results from various enzymatic modifications of skeleton molecules, these findings provide a great paradigm for building advanced isoflavonoid-producing microbial cell factories.

**FIGURE 1:**
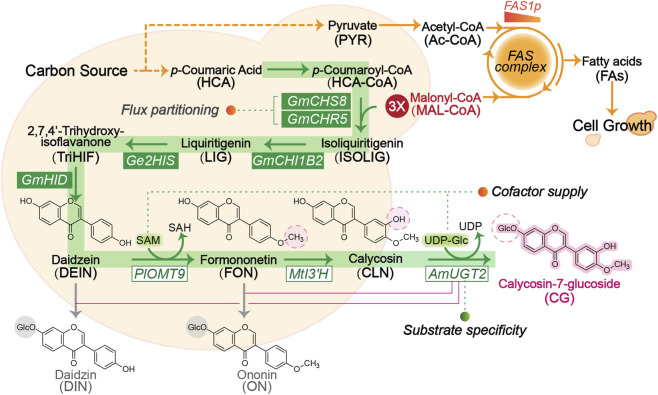
Engineering the complete biosynthesis of bioactive CG in yeast. A synthetic CG pathway was rebuilt and metabolically optimized by using a previously described precursor DEIN-producing strain. Yellow arrows, yeast native biosynthetic pathways, and flux toward fatty acids biosynthesis were fine-tuned to save more MAL-CoA for the CG route. Green arrows, introduced plant biosynthetic pathways, and the selected metabolic genes responsible for CG formation were highlighted in green boxes. Gray arrows, byproduct pathway. *GmCHS8*, chalcone synthase from *Glycine max*; *GmCHR5*, chalcone reductase from *G*. *max*; *GmCHI1B2*, chalcone isomerase from *G*. *max*; *Ge2HIS*, 2-hydroxyisoflavanone synthase from *Glycyrrhiza echinata*; *GmHID*, 2-hydroxyisoflavanone dehydratase from *G*. *max*; *PlOMT9*, O-methyltransferase from *Pueraria lobata*; *MtI3′H*, isoflavone-3′-hydroxylase from *Medicago truncatula*; *AmUGT2*, uridine diphosphate-dependent glucosyltransferase from *Astragalus membranaceus*. SAM, S-adenosylmethionine; SAH, S-adenosylhomocysteine; UDP-Glc, uridine diphosphate glucose; UDP, uridine diphosphate.

Here, we present the establishment of a yeast platform capable of *de novo* producing calycosin-7-glucoside (CG), a derivative of DEIN that comprises one of the quality markers of important medicinal herbs *Astragalus membranaceus* and *Astragalus mongholicus* Bunge (Tuan et al., 2015; Hao et al., 2023) (Figure 1). Through identifying active plant catalytic enzymes, optimizing carbon sources utilization, providing key precursor DEIN, and engineering biosynthesis of metabolic cofactors, production of 47.60 mg/L CG from simple carbon sources was achieved in shake flask fermentations. In addition, we showcased the feasibility of modifying the substrate preference of selected plant glycosyltransferases by rational protein engineering.

## 2 Materials and methods

### 2.1 Strains, plasmids, and reagents

*Escherichia coli* DH5α (Vazyme Bio) strain was used for the construction and amplification of plasmids, and BL21(DE3) (TransGen Bio) strain was used for protein expression. All plasmids and *S. cerevisiae* strains used in this study are listed in Supplementary Table S2 and Supplementary Table S5, respectively. SapphireAmp® Fast PCR Master Mix and PrimeStar DNA polymerase were purchased from TaKaRa Bio. One Step Cloning Kit, plasmid miniprep, DNA gel purification kits, and restriction enzymes were purchased from Vazyme Bio. All codon-optimized plant genes were chemically synthesized by GenScript Bio and are listed in Supplementary Table S4. All primers (Supplementary Table S6) were chemically synthesized by Sangon Bio. All chemicals and analytical standards were purchased from Shanghai Yuanye Bio.

### 2.2 Strain cultivation

YPD medium, consisting of 20 g/L peptone (Sangon Bio), 10 g/L yeast extract (Sangon Bio), and 30 g/L glucose (Shanghai Yuanye Bio), was used for routine yeast cultivation and preparation of competent cells. Synthetic complete medium without uracil (SC-URA), consisting of 6.7 g/L yeast nitrogen base (YNB) without amino acids (Formedium), 0.77 g/L complete supplement mixture without uracil (CSM-URA, Formedium), 20 g/L glucose (Shanghai Yuanye Bio), and 20 g/L agar (Shanghai Yuanye Bio), was used to select yeast transformants harboring the *URA3* marker. To drop out the *URA3* marker, yeast cultures were selected against on SC with 5-fluoroorotic acid (SC+5-FOA) plates, containing 6.7 g/L YNB, 0.77 g/L complete supplement mixture, and 1 g/L 5-FOA.

Shake flask fermentations for the production of isoflavonoids were carried out in a defined minimal medium (Verduyn et al., 1992), which consisted of 5 g/L (NH4)_2_SO_4_, 14.4 g/L KH_2_PO_4_, 0.5 g/L MgSO_4_∙7H_2_O, pH 6.0), 30 g/L glucose, 2 mL/L trace metal (3.0 g/L FeSO_4_∙7H_2_O, 4.5 g/L ZnSO_4_∙7H_2_O, 4.5 g/L CaCl_2_∙2H_2_O, 0.84 g/L MnCl_2_∙2H_2_O, 0.3 g/L CoCl_2_∙6H_2_O, 0.3 g/L CuSO_4_∙5H_2_O, 0.4 g/L Na_2_MoO_4_∙2H_2_O, 1.0 g/L H_3_BO_3_, 0.1 g/L KI and 19.0 g/L Na_2_EDTA∙2H_2_O), and 1 mL/L vitamin solutions (0.05 g/L D-Biotin, 1.0 g/L D-Pantothenic acid hemicalcium salt, 1.0 g/L Thiamin-HCl, 1.0 g/L Pyridoxin-HCl, 1.0 g/L Nicotinic acid, 0.2 g/L 4-aminobenzoic acid, 25.0 g/L myo-Inositol) (Verduyn et al., 1992), supplemented with 60 mg/L uracil and 1 mM 5-aminolevulinic acid (5-ALA) if required.

Single colonies, with PCR-verified genetic modifications, were inoculated into 14 mL tubes with 1.5 mL fresh minimal medium and incubated at 30 °C with 220 rpm agitation overnight. For precursor-supplemented fermentation, precultures were diluted and transferred to 14 mL tubes containing 2 mL minimal medium (with 50 mg/L substrate DEIN, FON, or CLN added) to an initial optical density measured at 600 nm (OD_600_) of 0.1 and cultivated at 220 rpm, 30 °C for an additional 72 h. For a stock solution of isoflavonoid intermediates, 5 g/L DEIN and FON were dissolved in DMSO, and 5 g/L CLN were dissolved in absolute ethanol, which were subsequently diluted with minimal medium to the abovementioned proportion. For shake flask fermentations, precultures were then diluted and transferred to a 125 mL non-baffled flask containing 15 mL minimal medium (added 10 g/L galactose into the medium) to induce the transcription of genes under the control of *GALps* at an initial OD_600_ of 0.05 and cultivated at 220 rpm, 30 °C for 72 h.

### 2.3 Genetic manipulation

All constructed yeast strains were derived from the genetic background *S. cerevisiae* CEN.PK113-5D-derivative IMX581 (*MATa ura3-52 can1Δ::cas9-natNT2 TRP1 LEU2 HIS3*) (Mans et al., 2015). For gene overexpression, *in vitro*–assembled DNA constructs were integrated at target genomic loci by means of the *CRISPR/Cas9* system. For the amplification of native promoters, genes, and terminators, IMX581 and BY4741 genomic DNA, and plasmid pZZY25, pZZY28, pZZY29, pZZY30, pZZY31 (laboratory storage) served as the template. Plasmids or synthetic fragments were utilized as the DNA template to amplify optimized heterologous genes (Supplementary Table S2; Supplementary Table S4). PrimeSTAR HS polymerase was used for routine DNA fragment amplifications and *in vitro* fusion PCR for generating integration constructs. Functional expression modules were generated according to an overlapping extension PCR (OE-PCR) procedure (Zhou et al., 2012). All used integration cassettes are listed in Supplementary Table S3.

Specific chromosomal loci (Mikkelsen et al., 2012), enabling stable and high-level expression of heterologous genes, were selected as the recombination sites for integration of gene overexpression constructs. Construction of strain CN01 was used as an example to show the CRISPR/Cas9-mediated integration of *in vitro* assembled DNA constructs. Fragment M8 was generated through assembling DNA parts *XII-1 us*, *PRM5t*, *PlOMT9*, *SkGAL2p*, *SuGAL2p*, *MtI3′H*, *SPO1t*, *GAT2t*. In detail, the upstream homologous arm *XII-1 us* (P15/P16) was amplified from IMX581 genomic DNA. Terminators *PRM5t* (P81/P82), *SPO1t* (P83/P84), and *GAT2t* (P85/P86) were amplified from BY4741 genomic DNA. Promoters *SkGAL2p* (P48/P49) and *SuGAL2p* (P50/P51) were amplified from plasmid pZZY30 and pZZY31, respectively. The coding regions of *PlOMT9* (P93/P96) and *MtI3′H* (P110/P114) were amplified using the synthetic gene as the template. Fragment M9 was assembled by fusing the DNA parts of *SPO1t*, *GAT2t*, *AmUGT2*, *SeGAL2p,* and *XII-1 ds*. Specifically, coding regions of *AmUGT2* (P132/P136) were amplified from synthetic genes. Promoters *SeGAL2p* (P52/P53) were amplified from plasmid pZZY28. The downstream homologous arm *XII-1 ds* (P17/P18) was amplified from IMX581 genomic DNA. Subsequently, equimolar amounts of purified fragments M8 and M9 (100 ng/kb) with *XII-1* targeting gRNA vector pCR02 (∼300 ng) were mixed and co-transformed into DEIN-producing strain P02 using the lithium acetate-mediated yeast transformation protocol (Mans et al., 2015).

The resulting transformants were selected on SC-URA plates, and colony PCR using SapphireAmp® Fast PCR Master Mix was performed to identify correct integrants. For gene deletion, 2 µg of a double-stranded DNA fragment consisting of two 50 bp sequences homologous to the flanking sequences of target genes, serving as the homologous repair of the genome double-strand break introduced by the cleavage of Cas9 nuclease, were co-transformed with corresponding gRNA vectors. Likewise, resulting transformants were selected on SC-URA plates, and colony PCR using SapphireAmp® Fast PCR Master Mix was performed to identify correct deletants. For the construction of the *FAS1* promoter-substitution strains, the native promoter sequences of *FAS1* (from −90 to 0 bp) were replaced by promoter *BGL2p* using the *CRISPR/Cas9* system.

To obtain recombinant plasmids for heterologous gene overexpression, the target gene, which consisted of two 20 bp sequences homologous to the vector backbone, was amplified from a synthetic fragment and subsequently recombined *in vitro* with the backbone of the high-copy plasmid p426GPD using the One Step Cloning Kit. The resulting recombinant plasmids were verified by sequencing.

Potential gRNAs for selected gene/genomic locus were identified and ranked with CEN.PK113-7D genetic background using the free and open CRISPRdirect tool (http://crispr.dbcls.jp/) (Naito et al., 2015). All gRNA plasmids were constructed by a One Step Cloning Kit in which gRNA sequence-containing DNA parts were *in vitro* recombined with a pMEL10 vector backbone (Mans et al., 2015). Correct recombinant plasmids were then verified by sequencing.

### 2.4 Metabolite extraction and quantification

Isoflavonoids production was quantified by high-performance liquid chromatography (HPLC) (Liu et al., 2021). In detail, 0.5 mL of cell culture was mixed with an equal volume of absolute acetonitrile (100% v/v), vortexed thoroughly, and centrifuged at 13,000 × *g* for 10 min. The supernatant was stored at −20 °C until HPLC analysis. Quantification of isoflavonoids was performed on a Vanquish HPLC DAD (Thermo Fisher Scientific, Waltham, MA, United States) equipped with a Discovery HS F5 15 cm × 4.6 mm column (particle size 5 μm, Sigma-Aldrich, St. Louis, MO, United States) connected to a photodiode array (PDA) detector (270 nm). The column was kept at 30 °C, and metabolites from 10 µL of supernatants were separated. Samples were analyzed using a gradient method with two solvents: water with 0.05% trifluoroacetic acid (A) and acetonitrile (B). For LIG, DEIN, DIN, FON, ON, CLN, and CG detection, a flow rate of 1.0 mL/min was used. The program started with 10% of solvent B (0–0.5 min), and the fraction was then maintained at 10% (0.5–10 min), after which the fraction was increased linearly from 10% to 40% (10–16 min). The fraction was increased linearly from 40% to 50% (16–21 min); after that, the fraction was decreased from 50% to 10% (21–22 min). Finally, the fraction was maintained at 10% (22–23 min).

All compounds were analyzed at a wavelength of 270 nm. LIG was detected at 21.2 min, DEIN at 18.6 min, DIN at 15.2 min, FON at 21.2 min, ON at 17.5 min, CLN at 18.6 min, and CG at 15.6 min. Chromeleon was used for HPLC data collection. Compound identity was confirmed by comparing the UV absorbance spectra and retention times of the samples with authentic standards. A six-point calibration curve, ranging from 1.5625 mg/L to 50 mg/L, was generated for the quantification of these chemicals. The *R* ^*2*^ coefficient for the resulting calibration curve was at least 0.99. Quantitative analysis was carried out using Microsoft Excel.

### 2.5 Glycosyltransferase gene cloning and protein expression

The coding region of *AmUGT2* (P209/P210) was amplified from a synthetic gene and *in vitro* recombined with a pET28a (Thermo Fisher Scientific) vector backbone with the One Step Cloning Kit. After sequence verification, the recombinant plasmid was transformed into *E. coli* BL21(DE3) (TransGen Bio). The resulting strain was cultivated in Luria–Bertani (LB) medium supplemented with 50 μg/mL kanamycin at 37 °C to an OD_600_ of 0.6. Protein expression was induced by the addition of 0.1 mM isopropyl β-D-thiogalactoside (IPTG), and the culture was further cultivated at 16 °C for an additional 20 h. Mutants were constructed and expressed in *E. coli* BL21 (DE3) following the same procedures.

### 2.6 Purification of proteins

To purify the protein, the bacterial cells were harvested by centrifugation (8,000 rpm, 3 min, 4 °C) and resuspended in 15 mL of lysis buffer (50 mM NaH_2_PO_4_, 30 mM NaCl, 10 mM imidazole, pH 8.0). The cells were then disrupted by ultrasonication on ice for 10 min, and the lysate was clarified by centrifugation at 12,000 rpm for 45 min to remove the debris. The supernatant was purified using a Ni-NTA column (Proteinlso Ni-NTA Resin, TransGen Biotech), and the protein was concentrated using Amicon Ultra-15 Ultracel-30K centrifuge filters (Merck Millipore).

### 2.7 *In vitro* enzyme activity assay and product quantification

The function of *AmUGT2* and its mutants was characterized by co-incubating 15 µg purified protein, 0.1 mM substrate, and 0.5 mM UDPG in 100 µL of 50 mM Tris-HCl buffer (37 °C, pH 8.0, 2 h). Methanol was added to terminate the reaction and centrifuged at 15,000 rpm for 20 min. Supernatant was detected using an HPLC 1260 System (Agilent Technologies, Santa Clara, CA, United States) equipped with a Zorbax SB C18 25 cm × 9.4 mm column (Agilent Technologies, Santa Clara, CA, United States) connected to a PDA detector (254 nm). The column was kept at room temperature. Samples were analyzed using a gradient method with two solvents: water with 0.1% formic acid (A) and methanol (B). For FON, ON, CLN, and CG detection, a flow rate of 1.0 mL/min was used.

The program started with 20% of solvent B, and the fraction was then increased linearly from 20% to 60% (0–8 min), after which it was increased linearly from 60% to 100% (8–12 min). The fraction was maintained at 100% (12–16 min); after that, the fraction was decreased from 100% to 20% (16–17 min). Finally, the fraction was maintained at 20% (17–18 min).

All compounds were detected at a wavelength of 254 nm. FON was detected at 13.6 min, ON at 10.8 min, CLN at 11.8 min, and CG at 8.8 min. Openlab was used for HPLC data collection. Compound identity was confirmed by comparing the UV absorbance spectra and retention times of the samples with authentic standards.

## 3 Results

### 3.1 Establishing the *de novo* biosynthesis of CG

In plants, as part of isoflavonoid metabolism, CG biosynthesis is fulfilled by a three-step enzymatic process that performs particularly structural modifications of the well-characterized scaffold isoflavone daidzein (DEIN). Specifically, formononetin (FON) is immediately formed by the O-methyltransferase (*OMT*)-mediated 4′-OH-specific methylation of DEIN (Li et al., 2016). Subsequently, P450 isoflavone 3′-hydroxylase (*I3′H*) is responsible for hydroxylation at 3′-OH to convert FON to calycosin (CLN) (Liu et al., 2003). The last reaction engages UDP-glycosyltransferase (*UGT*) for further attachment of the glucosyl moiety at 7-OH to produce CG (Tuan et al., 2015; Hao et al., 2023) (Figure 1). Hence, screening plant biosynthetic enzymes represents the first step toward developing a yeast platform for producing CG.

**FIGURE 2:**
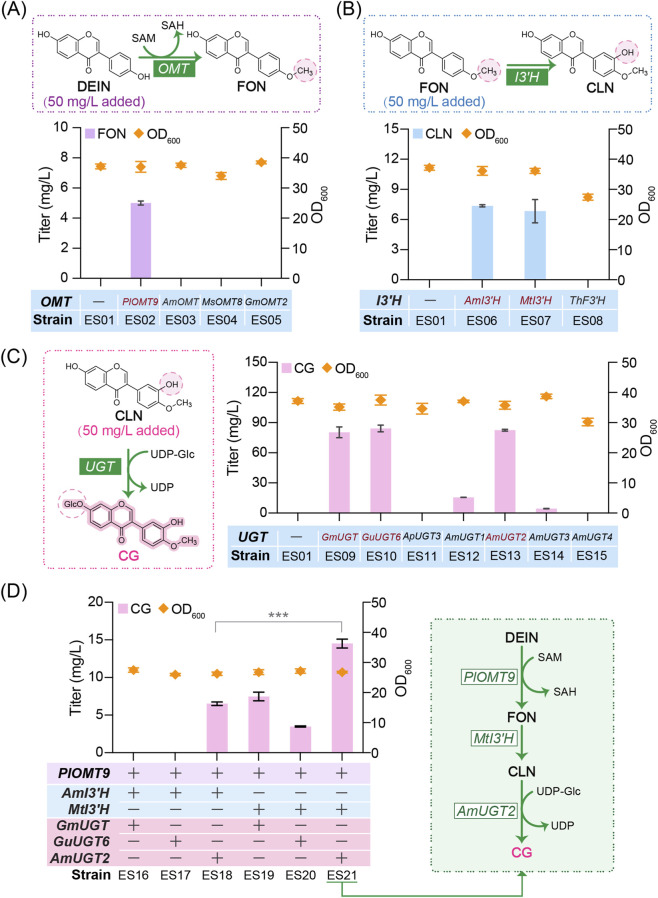
Building the biosynthetic pathway for CG. **(A–C)** Production profiles of FON, CLN, and CG produced by yeast strains harboring high-copy vector expressed plant OMTs, I3′Hs, and UGTs, respectively, with the presence of 50 mg/L corresponding substrates. **(D)** CG production of I15-derived strains overexpressing plant metabolic genes encoding selected OMT, I3′H, and UGT. For the source of selected plant genes: *Ms*, *Medicago sativa*; *Th*, *Torenia hybrid*; *Gu*, *Glycyrrhiza uralensis*; *Ap*, *Andrographis paniculata*. See Figure 1 legend regarding abbreviations of other plant species. Tube fermentations **(A–C)** were performed using a defined minimal medium with 30 g/L glucose as the sole carbon source. Shake flask fermentations **(D)** were performed using a defined minimal medium with 30 g/L glucose as the sole carbon source, and 1 mM 5-ALA and 10 g/L galactose were fed to amplify gene expression and activity of plant genes. All cultures were sampled after 72 h of growth for metabolite analysis. Statistical analysis was performed by using Student’s t*-*test (two-tailed; two-sample unequal variance; *p < 0.05, **p < 0.01, ***p < 0.001). All data represent the mean of *n* = 3 biologically independent samples, and error bars show standard deviation.

To rapidly identify active plant elements, we implemented *in vivo* activity assay for relevant plant candidate genes separately by feeding corresponding substrates. Four OMT-coding genes, including *PlOMT9* (*Pueraria lobata*), *AmOMT* (*Astragalus membranaceus*), *MsOMT8* (*Medicago sativa*), and *GmOMT2* (*Glycine max*), were selected and cloned into high-copy vector p426GPD under the control of strong constitutive promoter *TDH3p*, generating pCG01-04. Subsequently, these plasmids were transformed into the background strain IMX581, and single colonies were picked for further analysis. With the presence of 50 mg/L DEIN, only strain ES02 harboring the *PlOMT9* gene was able to produce a moderate amount of FON under shake flask cultivation (5.00 mg/L, Figure 2A). Similarly, out of three tested I3′H-coding candidate genes, *AmI3′H* from *A. membranaceus* and *MtI3′H* from *Medicago truncatula* exhibited detectable activity toward FON, whereas no CLN production was observed for strain ES08 expressing *ThF3′H* (*Torenia hybrid*) (Figure 2B). In search for effective plant UGTs, seven candidate genes were evaluated for their activities converting CLN to CG. Among all resultant strains (ES09-15, Figure 2C), comparable levels of CG (80.43–84.19 mg/L) were produced by strains ES09, ES10, and ES13, in which *GmUGT* (*G*. *max*), *GuUGT6* (*Glycyrrhiza uralensis*), and *AmUGT2* (*A. membranaceus*) were overexpressed, respectively. All these results provide the necessary plant catalytic enzymes for establishing microbial production of CG.

To facilitate the engineering of *de novo* complete biosynthesis of CG, we used a yeast platform strain (I15) that has been previously demonstrated to generate a moderate level of DEIN (34.3 mg/L) from glucose with the addition of galactose and 5-aminolevulinic acid (5-ALA) to amplify gene expression and activity of key P450 (Liu et al., 2021). Again, high-copy vectors carrying different combinations of selected plant genes, including 1 *OMT* (*PlOMT9*), 2 *I3′Hs* (*AmI3′H* and *MtI3′H*), and 3 *UGTs* (*GmUGT*, *GuUGT6,* and *AmUGT2*), were constructed and transformed into strain I15. Inducible promoters *GALp* were employed to transcribe these genes to align with the genetic characteristics of a DEIN-producing strain. Co-overexpression of *PlOMT9*, *MtI3′H,* and *AmUGT2* resulted in the best CG production at a level of 14.51 mg/L (strain ES21, Figure 2D). In addition, strains ES18-20 generated lower levels of CG (3.48–7.47 mg/L, Figure 2D). These data show the feasibility of reconstituting *de novo* biosynthesis of CG in yeast by exploiting plant metabolic diversity.

### 3.2 Optimizing carbon sources and DEIN biosynthesis to increase CG production

The type of carbon source has a profound impact on profiles of gene expression and cellular growth in *S. cerevisiae*. For our engineered CG-producing strains, galactose-induced dynamic transcription machinery was employed to guarantee sufficient expression of plant genes responsible for DEIN formation and subsequent conversion (Figure 1). In this context, initiation of *GALps*-mediated gene transcription can only occur when the preferred carbon source glucose is depleted (Egriboz et al., 2013; Ryo et al., 2017) due to the presence of catabolite repression and the Crabtree effect (Yu et al., 2018), which decouples CG formation from rapid cell growth. To mitigate the possible adverse role of this intrinsically metabolic regulation in CG biosynthesis, we therefore set out to evaluate alternative carbon sources in the fermentation of engineered yeast strains.

**FIGURE 3:**
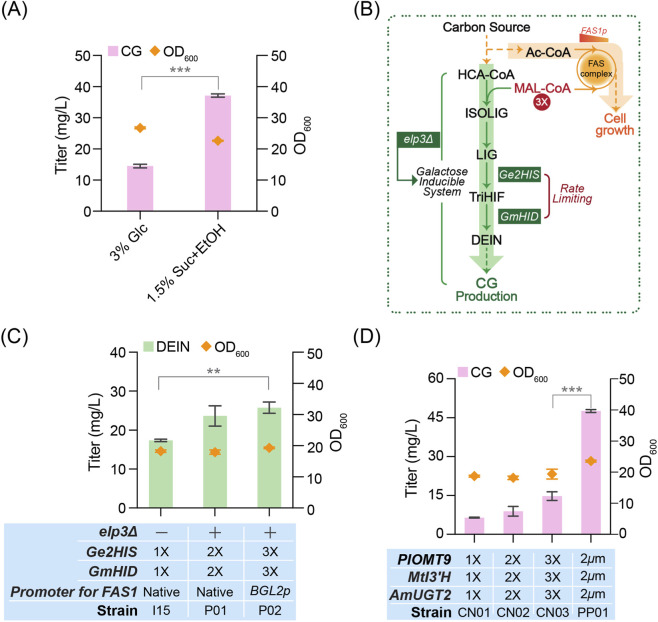
Optimizing carbon sources and DEIN biosynthesis to increase CG production. **(A)** Effect of different carbon sources on the CG production of strain ES21. Glc, glucose; Suc, sucrose; EtOH, ethanol. **(B)** Schematic illustration of the biosynthetic pathways leading to the production of DEIN and CG. Green arrows, designated DEIN and CG biosynthetic pathways. Yellow arrows, branching reactions for generating fatty acids. Expression of *FAS1*, encoding the beta subunit of the yeast fatty acid synthetase complex, was downregulated via promoter replacement. Deletion of *ELP3*, encoding a subunit of yeast elongator, and the RNAPII holoenzyme was carried out to reinforce the transcriptional activity of the *GAL* promoter. See Figure 1 and its legend regarding abbreviations of metabolites and gene details. **(C)** Production of precursor DEIN was increased through optimization of gene expression and yeast metabolic flux. **(D)** Production of CG by yeast strains carrying chromosomally integrated or plasmid-based plant genes. Shake flask fermentations were performed using a defined minimal medium with 15 g/L sucrose and 15 g/L ethanol as the carbon source, and 1 mM 5-ALA and 10 g/L galactose were fed to amplify gene expression and activity of plant genes. Cultures were sampled after 72 h of growth for metabolite analysis. Statistical analysis was performed by using Student’s t*-*test (two-tailed; two-sample unequal variance; *p < 0.05, **p < 0.01, ***p < 0.001). All data represent the mean of *n* = 3 biologically independent samples, and error bars show standard deviation.

A set of frequently used carbon sources, including glucose, sucrose, and ethanol, was first compared for DEIN production by strain I15 in shake flask cultivations. As expected, the addition of a mixture of 1.5% sucrose and 1.5% ethanol improved DEIN titer to 17.37 mg/L, a 35% increase relative to that of the standard 3% glucose condition (Supplementary Figure S1), while comparable growth profiles of I15 were observed for both cultivation conditions. Using this approach, CG generation of strain ES21 was significantly enhanced to 37.15 mg/L, accounting for a 156% increase relative to that obtained with 3% glucose addition (Figure 3A). These data indicate that a mixed carbon source could favor *GALps*-controlled biosynthesis of plant isoflavonoids and yeast cell growth simultaneously.

Based on the selection and evaluation of carbon sources, we speculated that the provision of DEIN could become a limiting factor for CG generation. We therefore tried to address this metabolic barrier by strengthening the expression level of introduced plant catalytic elements (Figure 3B). Previously, a loss-of-function mutation of Elp3, a histone acetyltransferase that acts as a subunit of the elongator and RNAPII holoenzyme in yeast, was demonstrated to reinforce the transcriptional activity of the *GAL* promoter (Wittschieben et al., 1999; Liu et al., 2021). Correspondingly, replacement of the *ELP3* gene with a second copy of *Ge2HIS* (*Glycyrrhiza echinata*) and *GmHID* (*G. max*) resulted in a further 36% (23.65 mg/L, strain P01) increase in DEIN titer over the control strain I15 (Figure 3C). Moreover, we rediverted central carbon flux toward the DEIN route by regulating the availability of malonyl-CoA (MAL-CoA), a key primary metabolite participating in both isoflavonoid metabolism and cellular growth. In budding yeast, most of the cytosolic malonyl-CoA pool is consumed for creating lipidic molecules, in which the FAS complex plays a role of metabolic valve (Wenz et al., 2001) (Figure 3B). To save more intracellular MAL-CoA for DEIN biosynthesis in strain P01, expression of the *FAS1* gene, which encodes a subunit of the FAS complex, was downregulated by replacing the endogenous *FAS1p* with a weaker counterpart, *BGL2p* (Liu et al., 2021). Similarly, an additional copy of *Ge2HIS* and *GmHID* was integrated as well. These modifications further boosted DEIN production to a titer of 25.77 mg/L (strain P02), representing an 48% increase compared with that of strain I15 (Figure 3C).

With the established strains capable of generating elevated levels of DEIN, we had an interest in rebuilding complete CG biosynthesis enabled by chromosomally integrated plant pathway, which is believed to possess a greater degree of genetic and phenotypic stability compared with plasmid-involved bioproduction. Based on the best DEIN-generating strain P02, three strains, CN01-03, harboring varied copy numbers of selected CG-forming genes (*PlOMT9*, *MtI3′H*, and *AmUGT2*), were constructed. Product analysis showed that the titer of CG kept increasing as more downstream plant genes were integrated, and strain CN03 accumulated the highest level of CG to 14.75 mg/L (Figure 3D). Interestingly, production of CG could notably increase to 47.60 mg/L by strain PP01, in which a high-copy vector was introduced for gene overexpression, and this value represents a 2.23-fold increase compared with strain CN03 (Figure 3D). These results clearly illustrate that the low catalytic activity of heterologous enzymes is rate-limiting in CG production, as evidenced by the accumulation of intermediates and buildup of byproducts in strains CN03 and PP01, especially the significant generation of glycosides ononin (ON, 12.70 mg/L by strain CN03) and daidzin (DIN, 15.85 mg/L by strain PP01) (Supplementary Figure S2).

### 3.3 Engineering metabolic cofactors related to CG biosynthesis

**FIGURE 4:**
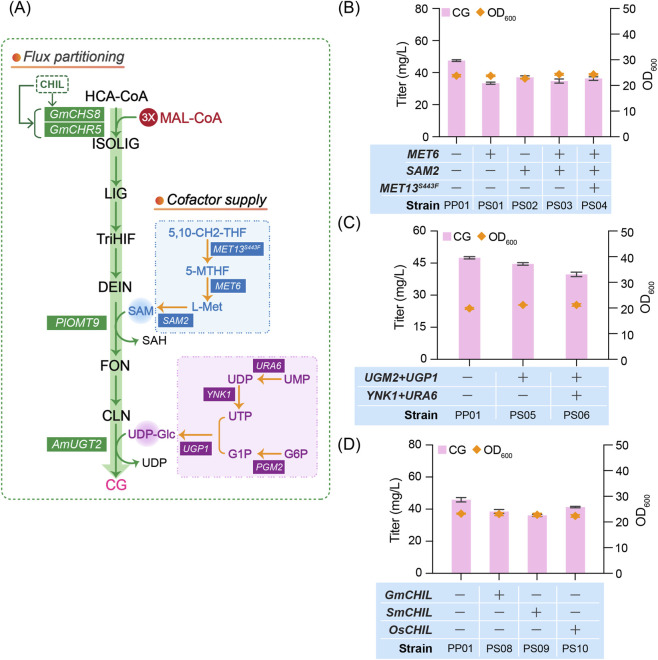
Investigation of metabolic factors affecting CG biosynthesis. **(A)** Schematic illustration of the biosynthesis of relevant metabolic cofactors for CG biosynthesis. Green box, plant flux partitioning mechanism regulating the formation of scaffold ISOLIG. Blue box, endogenous biosynthetic pathway of the methyl donor SAM. Magenta box, endogenous biosynthetic pathway of the glycosyl donor UDP-Glc. CHIL, plant chalcone isomerase-like protein; *MET13* ^*S443F*^, mutant of methylenetetrahydrofolate reductase; *MET6*, methionine synthetase; *SAM2*, methionine adenosyltransferase; *URA6*, uridylate kinase; *YNK1*, nucleoside diphosphate kinase; *PGM2*, phosphoglucomutase; *UGP1*, uridine diphosphate glucose pyrophosphorylase. 5,10-CH2-THF, 5,10-methylenetetrahydrofolic acid; 5-MTHF, 5-methenyltetrahydrofolic acid; L-Met, L-methionine; UMP, uridine monophosphate; UTP, uridine triphosphate; G6P, glucose-6-phosphate; G1P, glucose-1-phosphate. See Figure 1 and its legend regarding abbreviations of other metabolites and gene details. Effect of enhanced anabolism of SAM **(B)** and UDP-Glc **(C)**, and introduction of plant-derived metabolic regulatory factors **(D)** on the production of CG. For the source of selected plant genes: *Sm*, *Selaginella moellendorffii*; *Os*, *Oryza sativa*. See Figure 1 legend regarding abbreviations of other plant species. Shake flask fermentations were performed using a defined minimal medium with 15 g/L sucrose and 15 g/L ethanol as the carbon source, and 1 mM 5-ALA and 10 g/L galactose were fed to amplify gene expression and activity of plant genes. Cultures were sampled after 72 h of growth for metabolite analysis. Cultures were sampled after 72 h of growth for metabolite analysis. All data represent the mean of *n* = 3 biologically independent samples, and error bars show standard deviation.

Bioconversion of DEIN to CG involves three sequential enzymatic reactions that all require the investment of specific metabolic cofactors (Figure 1). As systematic engineering of NADPH production has been implemented to optimize the performance of plant P450s introduced in our initial DEIN-generation strain I15 (Liu et al., 2021), we next explored the effect of enhanced supply of other metabolic cofactors, including S-adenosylmethionine (SAM) and uridine diphosphate glucose (UDP-Glc), on CG production (Figure 4A).

To reroute the metabolic flux toward generating SAM, essential for the activity of O- methyltransferase *PlOMT9*, different genetic modifications were devised and implemented in DEIN-producing strain P02, and the high-copy vector pCG20 was subsequently transformed for CG formation analysis. In the first strategy, individual overexpression of native metabolic enzymes was performed for methionine synthase Met6, which catalyzes the conversion of L-homocysteine to L-methionine, and *SAM2*-encoded ATP-dependent methionine adenosyltransferase, which directly converts L-methionine to SAM (Lv et al., 2024). The resultant strains PS01 and PS02 produced 33.42 and 37.04 mg/L of CG, representing a 30% and 22% decrease, respectively, compared with the control strain PP01 (Figure 4B). Moreover, combined overexpression of these two enzymes exhibited a similar impact on CG production (strain PS03, Figure 4B). In addition, activity of methylenetetrahydrofolate reductase Met13 is reported to be negatively feedback-regulated by SAM, and expression of its mutant allele Met13^S443F^ has been demonstrated to significantly increase the abundance of L-methionine (Isogai et al., 2024). We therefore further included this genetic modification, and no improvement occurred in the CG biosynthesis by the resultant strain PS04 (Figure 4B).

Parallelly, we enhanced the capacity of strain P02 for biosynthesizing UDP-glucose, a metabolic cofactor that provides the glycosyl group donor for glycosyltransferase AmUGT2. In budding yeast, the formation of UDP-glucose is directly catalyzed by UDP-glucose pyrophosphorylase (encoded by UGP1), in which one molecule of glucose-1-phosphate (G1P) and uridine triphosphate (UTP) are consumed (Crowe et al., 2024). As to these two precursors, phosphoglucomutase Pgm2 is responsible for the conversion of glucose-6-phosphate (G6P) to G1P, whereas a two-step reaction catalyzed by uridine kinase Ura6 and nucleoside diphosphate kinase Ynk1 leads to production of UTP from uridine monophosphate (UMP) (Ma et al., 1990; Fukuchi et al., 1993). Through chromosomally integrated expression of these key enzymes and the introduction of multi-copy vector pCG20, strains PS05 and PS06 were created, which produced a slightly decreased level of CG at 44.62 and 39.72 mg/L, respectively (Figure 4C). Conversely, the titer of CG was further compromised to 26.16 mg/L by strain PS07 harboring combined engineering of SAM and UDP-Glc anabolism (Supplementary Figure S3). These results indicate that metabolic cofactors may not play a role-limiting factor for CG biosynthesis, at least so far.

Besides optimization of yeast endogenous anabolism, we turned to capture plant-derived metabolic regulatory mechanisms to improve CG generation (Figure 4A). Previously, plant isoflavonoid metabolism has been characterized to be *in situ* regulated by chalcone isomerase-like proteins (CHILs) (Ni et al., 2020), which could interact with chalcone synthase and thereby reduce metabolic flux toward byproduct formation. Subsequently, overexpression of *GmCHIL* (*G. max*) proved to be able to significantly enhance DEIN production, accompanied by decreased buildup of byproducts bis-noryangonin and *p*-coumaroyltriacetic acid lactone in *S. cerevisiae* (Raytek et al., 2025). Therefore, a set of CHIL-coding genes from different plant origins, including *GmCHIL*, *SmCHIL* (*Selaginella moellendorffii*), and *OsCHIL* (*Oryza sativa*), was individually overexpressed in strain P02. However, the reported synergistic effect was not observed regarding DEIN and CG production, and for the latter *OsCHIL*-expressing strain, PS10 accumulated the best CG titer at 41.30 mg/L, accounting for a 10% decrease relative to the control PP01 strain (Figure 4D; Supplementary Figure S4). Collectively, these results imply that the overall performance of CG-generating plant metabolic enzymes needs to be optimized to render more efficient CG biosynthesis.

### 3.4 Improving substrate preference of plant UGT through rational protein engineering

**FIGURE 5:**
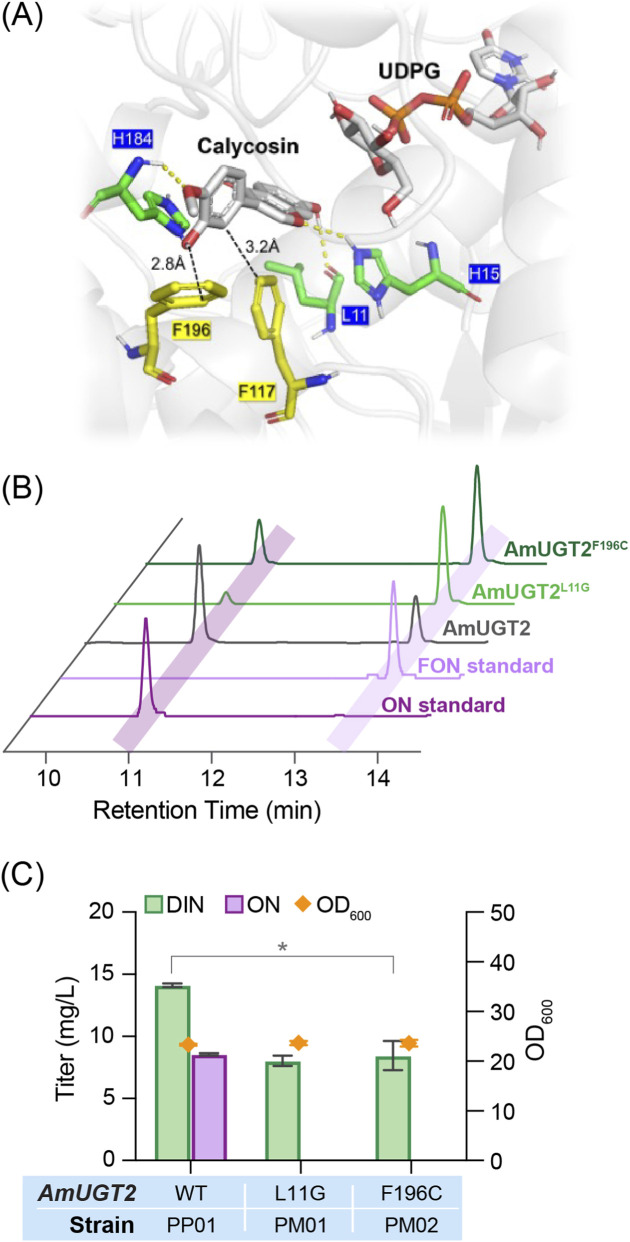
Improving the substrate preference of plant UGT through rational protein engineering. **(A)** Schematic illustration of the molecular docking simulation of CLN and UDP-Glc in the predicted structure of AmUGT2. Blue shading, amino acid residues that form hydrogen bonds with CLN. Yellow shading, amino acid residues that cause steric hindrance with CLN. **(B)** *In vitro* enzyme activity assay of AmUGT2 and its mutants using FON as substrate. Peak area indicates the amount of the substance at the shown retention time. **(C)** Production profiles of DIN and ON produced by yeast strains expressing *AmUGT2* and its mutants. Shake flask fermentations were performed using a defined minimal medium with 15 g/L sucrose and 15 g/L ethanol as the carbon source, and 1 mM 5-ALA and 10 g/L galactose were fed to amplify gene expression and activity of plant genes. Cultures were sampled after 72 h of growth for metabolite analysis. Cultures were sampled after 72 h of growth for metabolite analysis. Statistical analysis was performed by using Student’s t*-*test (two-tailed; two-sample unequal variance; *p < 0.05, **p < 0.01, ***p < 0.001). All data represent the mean of *n* = 3 biologically independent samples, and error bars show standard deviation.

Directed evolution represents a powerful tool for rapidly boosting the catalytic properties of enzymes of interest with respect to both catalytic activity and substrate specificity (Turner, 2009). The substantial accumulation of glycosides ON and DIN in the best CG-producing strain PP01 (Supplementary Figure S2), a waste of precursor DEIN that can be attributed to the reported broad substrate recognition by glycosyltransferase AmUGT2, prompted us to take action to confront this metabolic limitation. In doing so, we first employed AlphaFold3 for structure prediction of AmUGT2, with reference to the known structure of GgCGT from *Glycyrrhiza glabra* (PDB ID: 6L5P) (Zhang et al., 2020), and the sugar donor UDP-Glc was embedded into the predicted structure. Subsequently, the target acceptor molecule CLN was docked into the active pocket of AmUGT2 using Autodock (Figure 5A). Molecular docking results revealed that CLN formed hydrogen bonds with amino acid residues L11, H184, and H15 of AmUGT2. Specifically, L11 formed a hydrogen bond with the 7-OH of CLN, whereas H184 simultaneously formed hydrogen bonds with the 3′-OH and the oxygen atom of the 4′-OCH_3_ group of CLN. However, L11 and H184 are positioned on the opposite side of CLN relative to the sugar donor UDP-Glc, which may impede the formation of a favorable reactive orientation between CLN and UDP-Glc. Meanwhile, amino acid residues F117 and F196 influence the positioning of CLN within the active pocket of AmUGT2 through steric hindrance and hydrophobic interactions, similarly restricting CLN from adopting an orientation more conducive to glycosylation. Based on these hypotheses, we constructed a rationally designed small mutant library targeting residues L11, H184, F117, and F196 of AmUGT2 (Supplementary Table S1), with the core strategy of eliminating non-productive hydrogen bonds and optimizing steric hindrance.

*In vitro* enzymatic activity assays were conducted for all available mutants using CLN and FON as substrates. HPLC analysis revealed that mutants L11G and F196C exhibited comparable catalytic activity toward CLN relative to the wild-type AmUGT2, with the majority of CLN being converted to CG (Supplementary Figure S5A). However, as to the consumption of competing substrate FON, a remarkably decreased conversion efficiency was observed for both L11G and F196C mutants, whereas the wild-type AmUGT2 enzyme produced a substantial amount of ON, as expected (Figure 5B). Moreover, *in vivo* catalytic performance of both mutants was evaluated for the production of CG and byproduct glycosides following the above genetic modification framework. Interestingly, AmUGT2 mutant-expressing strains PM01 and PM02 accumulated about 8.04 and 8.45 mg/L of DIN, representing a 43% and 40% decrease, respectively, compared with strain PP01, and no detectable level of ON was generated in these two strains (Figure 5C). Additionally, a titer of 38.20 and 40.48 mg/L of CG was produced by strains PM01 and PM02, representing 84% and 89% of that obtained by the control strain (Supplementary Figure S5B). Moreover, no significant improvement was observed for the remaining AmUGT2 mutants with respect to their *in vitro* enzymatic activity and specificity (Supplementary Figure S6). These findings indicate that these two single-point mutations (L11G and F196C) effectively enhanced the substrate preference of the selected glycosyltransferase.

## 4 Discussion

Development of efficient microbial cell factories represents the very first step toward the biomanufacturing of plant natural products, including isoflavonoids that have been found in many traditional Chinese medicinal herbs. Here, we established a yeast-based production platform for the efficient production of the isoflavonoid CG from renewable carbon sources. With the aid of a previously described precursor-generating strain I15 (Liu et al., 2021), we carried out the following metabolic engineering efforts step-wise: identifying plant functional enzymes and rebuilding the CG biosynthetic pathway, optimizing the type of carbon source and supply of precursor DEIN to increase CG titer, enhancing the anabolism of endogenous cofactors SAM and UDP-Glc, and improving the catalytic preference of selected plant UGT. The best strain, PP01, which was able to produce 47.60 mg/L of CG when a mixed carbon source (1.5% sucrose and 1.5% ethanol) was adopted for shake flask cultivations (Figure 3D). Notably, a very recent study independently demonstrated heterologous biosynthesis of CG in budding yeast as well, though a lower production of CG (0.22 mg/L) has been achieved (Chen et al., 2025). This significant discrepancy could result from the choice of genetic background, the inclusion of different plant catalytic elements, and differences in cultivation conditions.

Metabolic enzymes responsible for the biosynthesis of plant isoflavonoids usually exhibit significantly low catalytic activities as a result of natural evolution and selection (Bar-Even et al., 2011). While this deficiency remains a huge challenge at present to be overcome in generating desired microbial cell factories, there are several measures that could be routinely performed to relieve its adverse effect. The first strategy relates to amplifying the expression levels of plant candidate genes. Indeed, when CG biosynthetic genes were overexpressed from a multi-copy vector, a 223% improvement of CG titer was obtained compared with that of the strain harboring genomically integrated plant genes (Figure 3D). Moreover, various metabolic cofactors, such as NADPH, SAM, UDP-Glc, play an essential role in supporting the activity of CG-producing enzymes. We chose to enhance the supply of SAM and UDP-Glc in yeast; however, no improvement occurred as to CG generation, no matter which relevant native genes were overexpressed individually or combinatorially (Figure 4). Buildup of metabolic intermediates and byproducts is frequently observed for microbe-mediated biosynthesis of plant natural products (Liu et al., 2021; Payne et al., 2021), which can be reduced by enhancing the catalytic activity and selectivity of downstream pathway routes. For our engineered strains, a considerable amount of byproduct glycosides, including DEIN-derived DIN and FON-derived ON, was also generated (Supplementary Figure S2), which is consistent with a previous study of AmUGT2 (Hao et al., 2023). We then turned to explore the potential of rational protein engineering in boosting the catalytic performance of these important CG biosynthetic enzymes. Notably, significantly decreased formation of byproducts was demonstrated for two single-point mutants (L11G and F196C), while they exhibited comparable activity toward CG biosynthesis with that of the wild-type AmUGTs, as evidenced by both *in vitro* and *in vivo* analysis (Figure 5; Supplementary Figure S5).

In conclusion, as a proof-of-concept study, we have successfully reconstituted complete CG biosynthesis and turned the preferred workhorse *S. cerevisiae* into a novel platform for producing this important bioactive isoflavonoid. Though the resulting CG titers need to be greatly enhanced for commercial application, our work still provides insights for further engineering efforts. For example, novel plant metabolic enzymes may be readily evaluated using the established CG-producing strains. In addition, the development and application of biosensor-assisted selection approach (Skjoedt et al., 2016; Mitchler et al., 2021) should accelerate the performance evolution of enzymes governing the rate-limiting biochemical reactions and thereby further improve CG production by means of enabling superior titers and/or removal of byproduct formation (Liu et al., 2021).

## Data Availability

The datasets presented in this study can be found in online repositories. The names of the repository/repositories and accession number(s) can be found in the article/Supplementary Material.
