# Supplementary material for: Efficient, complete biosynthesis of isoflavonoid calycosin-7-glucoside by metabolically engineered yeast cell factories
Source: Front Bioeng Biotechnol. 2026 Jul 9;14:1875470. doi: 10.3389/fbioe.2026.1875470 (PMC13391940; doi:10.3389/fbioe.2026.1875470)
Supplement: Supplementary file 1 [file Supplementaryfile1.docx]

Supplementary Materials for

Efficient complete biosynthesis of isoflavonoid calycosin-7-glucoside by metabolically engineered yeast cell factories

Weifeng Zhang^1,2^, Mingyuan Xu^2,3,4^, Linlin Wang^5^, Xue Qiao^5^, Song Yang^1^, Yi Liu^2,3,4*^, Quanli Liu^2,3,4*^, Xuefeng Lu^2,3,4*^

^1^ School of Life Sciences, Qingdao Agricultural University, 700 Changcheng Road, Qingdao 266000, China.

^2^ State Key Laboratory of Photoelectric Conversion and Utilization of Solar Energy, Qingdao Institute of Bioenergy and Bioprocess Technology, Chinese Academy of Sciences, 189 Songling Road, Qingdao 266101, China

^3^ Shandong Energy Institute, 189 Songling Road, Qingdao 266101, China

^4^ University of Chinese Academy of Sciences, Beijing 100049, China.

^5^ State Key Laboratory of Natural and Biomimetic Drugs, School of Pharmaceutical Sciences, Peking University, 38 Xueyuan Road, Beijing 100191, China.

^*^ Correspondence:

Yi Liu

[liuyi@qibebt.ac.cn](mailto:liuyi@qibebt.ac.cn);

Quanli Liu

[liuql@qibebt.ac.cn](mailto:liuql@qibebt.ac.cn);

Xuefeng Lu

lvxf@qibebt.ac.cn


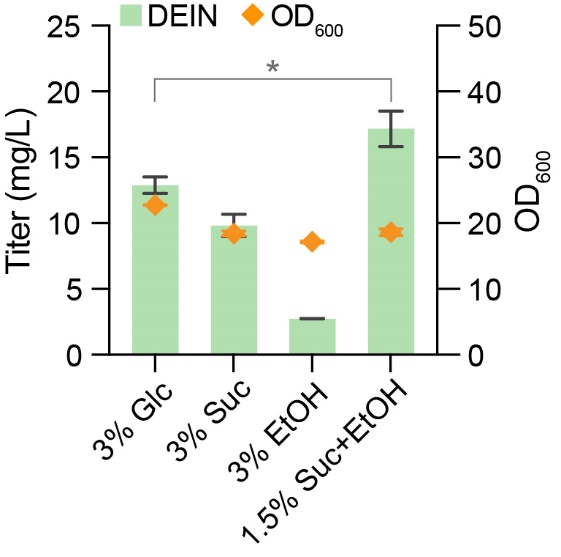


**Supplementary Figure S1 Effect of different carbon sources on the production of DEIN by strain I15.** Shake flask fermentations were performed using a defined minimal medium with 15 g/L sucrose and 15 g/L ethanol as the carbon source, and 1 mM 5-ALA and 10 g/L galactose were fed to amplify gene expression and activity of plant genes. Cultures were sampled after 72 h of growth for metabolite analysis. Statistical analysis was performed by using Student’s *t* test (two-tailed; two-sample unequal variance; **p* < 0.05, ***p* < 0.01, ****p* < 0.001). All data represent the mean of *n* = 3 biologically independent samples and error bars show standard deviation.


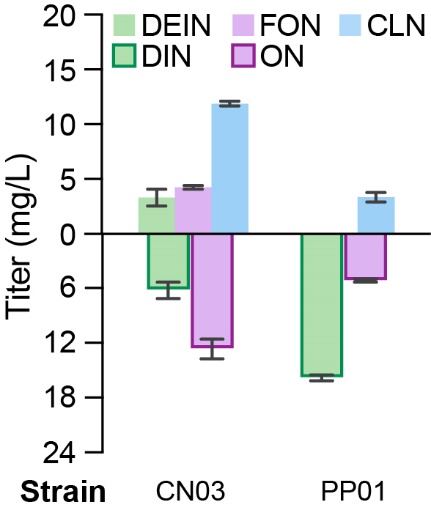


**Supplementary Figure S2 Residual of intermediates and accumulation of byproduct glycosides in CG-producing strains.** Shake flask fermentations were performed using a defined minimal medium with 15 g/L sucrose and 15 g/L ethanol as the carbon source, and 1 mM 5-ALA and 10 g/L galactose were fed to amplify gene expression and activity of plant genes. Cultures were sampled after 72 h of growth for metabolite analysis. All data represent the mean of *n* = 3 biologically independent samples and error bars show standard deviation.


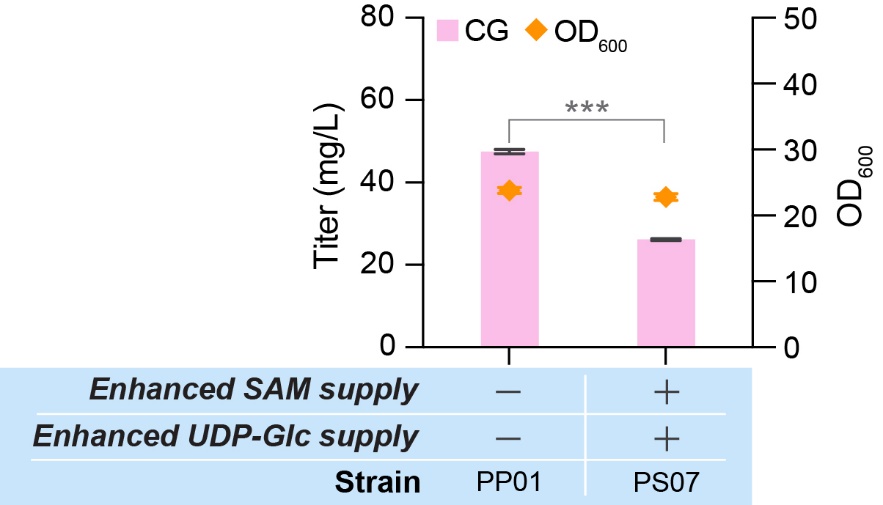


**Supplementary Figure S3 Effect of simultaneously enhanced SAM and UDP-Glc supply on the production of CG.** Shake flask fermentations were performed using a defined minimal medium with 15 g/L sucrose and 15 g/L ethanol as the carbon source, and 1 mM 5-ALA and 10 g/L galactose were fed to amplify gene expression and activity of plant genes. Cultures were sampled after 72 h of growth for metabolite analysis. Statistical analysis was performed by using Student’s *t* test (two-tailed; two-sample unequal variance; **p* < 0.05, ***p* < 0.01, ****p* < 0.001). All data represent the mean of *n* = 3 biologically independent samples and error bars show standard deviation.


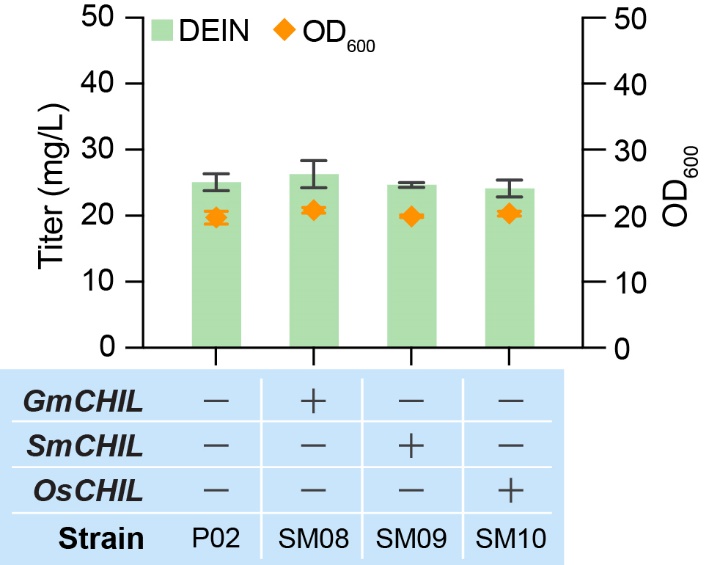


**Supplementary Figure S4 Effect of plant CHILs overexpression on the production of DEIN.** Shake flask fermentations were performed using a defined minimal medium with 15 g/L sucrose and 15 g/L ethanol as the carbon source, and 1 mM 5-ALA and 10 g/L galactose were fed to amplify gene expression and activity of plant genes. Cultures were sampled after 72 h of growth for metabolite analysis. All data represent the mean of *n* = 3 biologically independent samples and error bars show standard deviation.


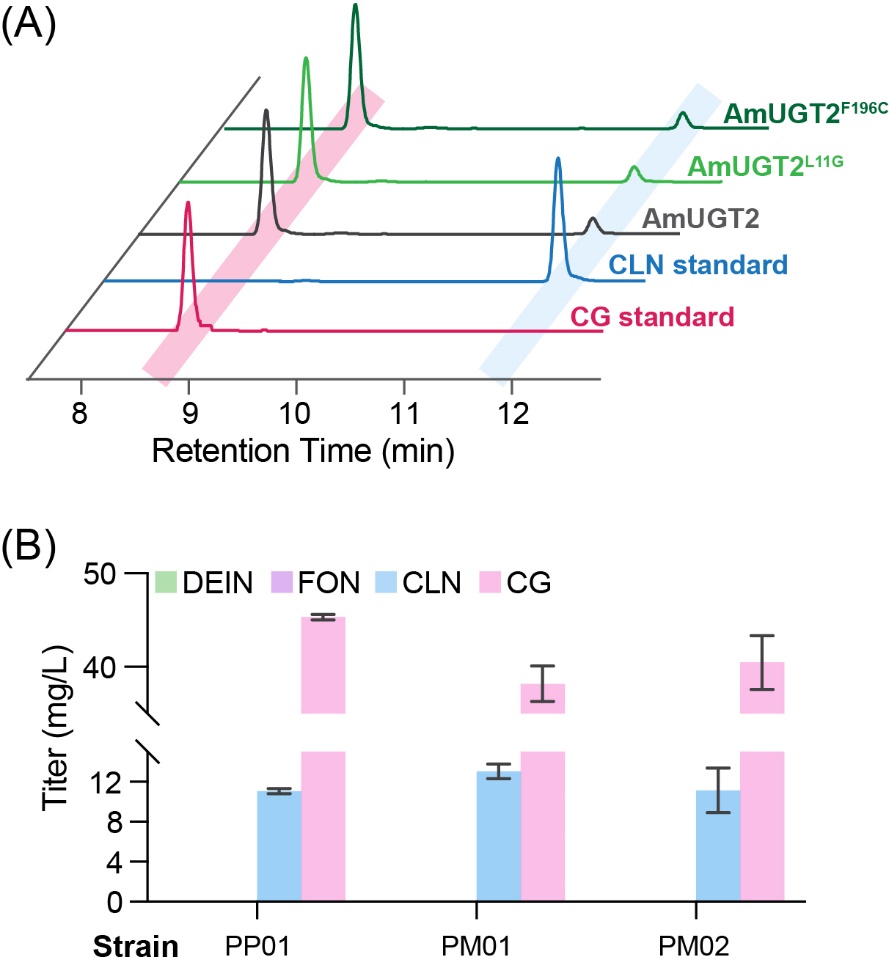


**Supplementary Figure S5 Performance of AmUGT2 and its mutants towards production of relevant glycosides.** (A) *In vitro* enzyme activity assay of AmUGT2 and its mutants using CLN as substrate. Peak area indicates the amount of the substance at the given retention time. (B) Production profiles of CG produced by yeast strains expressing AmUGT2 and its mutants. Shake flask fermentations were performed using a defined minimal medium with 15 g/L sucrose and 15 g/L ethanol as the carbon source, and 1 mM 5-ALA and 10 g/L galactose were fed to amplify gene expression and activity of plant genes. Cultures were sampled after 72 h of growth for metabolite analysis. All data represent the mean of *n* = 3 biologically independent samples and error bars show standard deviation.


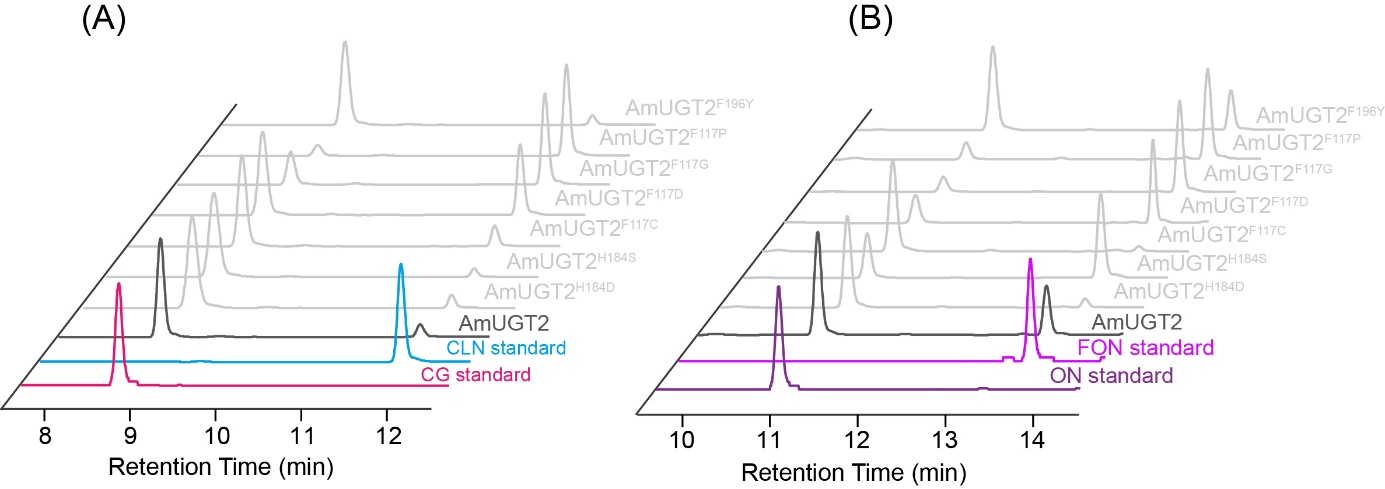


**Supplementary Figure S6 *In vitro* enzyme activity assay of AmUGT2 and its mutants using CLN (A) and** **FON (B) as the substrate, respectively.** Peak area indicates the amount of the substance at the shown retention time.

**Supplementary Table S1 Rationally designed mutant library of AmUGT2.**

| **No.** | **Amino acid mutation** |
| --- | --- |
| 1 | L11G |
| 2 | H184D |
| 3 | H184S |
| 4 | F117C |
| 5 | F117D |
| 6 | F117G |
| 7 | F117P |
| 8 | F196C |
| 9 | F196Y |

**Supplementary Table S2 Plasmids used in this study.**

| **Plasmid ID** | **Relevant characteristics** | **Origin** |
| --- | --- | --- |
| **Template plasmids** | | |
| pUC19 | *pUC Ori ampR* | Lab store |
| pZZY25 | *pUC Ori ampR* template for *SkGAL1p* | Lab store |
| pZZY28 | *pUC Ori ampR* template for *SeGAL2p* | Lab store |
| pZZY29 | *pUC Ori ampR* template for *SmGAL2p* | Lab store |
| pZZY30 | *pUC Ori ampR* template for *SkGAL2p* | Lab store |
| pZZY31 | *pUC Ori ampR* template for *SuGAL2p* | Lab store |
| pAS41 | *pUC Ori ampR* template for ***MET13*** | This study |
| pAS42 | *pUC Ori ampR* template for ***MET13^S443F^*** | This study |
| **Gene overexpression** | | |
| p426GPD | *2μm ampR URA3 TDH3p-CYC1t* | Lab store |
| pCG01 | *2μm ampR URA3 TDH3p-****PlOMT9****-CYC1t* | This study |
| pCG02 | *2μm ampR URA3 TDH3p-****AmOMT****-CYC1t* | This study |
| pCG03 | *2μm ampR URA3 TDH3p-****MsOMT8****-CYC1t* | This study |
| pCG04 | *2μm ampR URA3 TDH3p-****GmOMT****-CYC1t* | This study |
| pCG05 | *2μm ampR URA3 TDH3p-****AmI3'H****-CYC1t* | This study |
| pCG06 | *2μm ampR URA3 TDH3p-****MtI3'H****-CYC1t* | This study |
| pCG07 | *2μm ampR URA3 TDH3p-****ThF3'H****-CYC1t* | This study |
| pCG08 | *2μm ampR URA3 TDH3p-****GmUGT****-CYC1t* | This study |
| pCG09 | *2μm ampR URA3 TDH3p-****GuUGT6****-CYC1t* | This study |
| pCG10 | *2μm ampR URA3 TDH3p-****ApUGT3****-CYC1t* | This study |
| pCG11 | *2μm ampR URA3 TDH3p-****AmUGT1****-CYC1t* | This study |
| pCG12 | *2μm ampR URA3 TDH3p-****AmUGT2****-CYC1t* | This study |
| pCG13 | *2μm ampR URA3 TDH3p-****AmUGT3****-CYC1t* | This study |
| pCG14 | *2μm ampR URA3 TDH3p-****AmUGT4****-CYC1t* | This study |
| pCG15 | *2μm ampR URA3 CYC1t-****PlOMT9****-GAL1p, GAL7p-****AmI3'H****-HIS5t, FBA1t-****GmUGT****-GAL10p* | This study |
| pCG16 | *2μm ampR URA3 CYC1t-****PlOMT9****-GAL1p, GAL7p-****AmI3'H****-HIS5t, FBA1t-****GuUGT6****-GAL10p* | This study |
| pCG17 | *2μm ampR URA3 CYC1t-****PlOMT9****-GAL1p, GAL7p-****AmI3'H****-HIS5t, FBA1t-****AmUGT2****-GAL10p* | This study |
| pCG18 | *2μm ampR URA3 CYC1t-****PlOMT9****-GAL1p, GAL7p-****MtI3'H****-HIS5t, FBA1t-****GmUGT****-GAL10p* | This study |
| pCG19 | *2μm ampR URA3 CYC1t-****PlOMT9****-GAL1p, GAL7p-****MtI3'H****-HIS5t, FBA1t-****GuUGT6****-GAL10p* | This study |
| pCG20 | *2μm ampR URA3 CYC1t-****PlOMT9****-GAL1p, GAL7p-****MtI3'H****-HIS5t, FBA1t-****AmUGT2****-GAL10p* | This study |
| pCG21 | *2μm ampR URA3 CYC1t-****PlOMT9****-GAL1p, GAL7p-****MtI3'H****-HIS5t, FBA1t-****AmUGT2^L11G^****-GAL10p* | This study |
| pCG22 | *2μm ampR URA3 CYC1t-****PlOMT9****-GAL1p, GAL7p-****MtI3'H****-HIS5t, FBA1t-****AmUGT2^F196C^****-GAL10p* | This study |
| **gRNA vectors** | | |
| pMEL10 | *2μm ampR KlURA3 gRNA-CAN1.Y* | Lab store |
| pCR01 | *2μm ampR KlURA3 gRNA-****XI-1****.Y* | This study |
| pCR02 | *2μm ampR KlURA3 gRNA-****XII-1****.Y* | This study |
| pCR03 | *2μm ampR KlURA3 gRNA-****XII-3****.Y* | This study |
| pQC006 | *2μm ampR KlURA3 gRNA-****XI-3****.Y* | Lab store |
| pQC010 | *2μm ampR KlURA3 gRNA-****XII-4****.Y* | Lab store |
| pQC0197 | *2μm ampR KlURA3 gRNA-****FAS1p****.Y [2×]* | Lab store |
| pQC0238 | *2μm ampR KlURA3 gRNA-****ELP3****.Y* | Lab store |
| **Protein expression** | | |
| pET28a | *pBR322 Ori kanR* | Lab store |
| pPE01 | *pBR322 Ori kanR T7p-****AmUGT2****-T7t* | This study |
| pPE02 | *pBR322 Ori kanR T7p-****AmUGT2^L11G^****-T7t* | This study |
| pPE03 | *pBR322 Ori kanR T7p-****AmUGT2^H184D^****-T7t* | This study |
| pPE04 | *pBR322 Ori kanR T7p-****AmUGT2^H184S^****-T7t* | This study |
| pPE05 | *pBR322 Ori kanR T7p-****AmUGT2^F117C^****-T7t* | This study |
| pPE06 | *pBR322 Ori kanR T7p-****AmUGT2^F117D^****-T7t* | This study |
| pPE07 | *pBR322 Ori kanR T7p-****AmUGT2^F117G^****-T7t* | This study |
| pPE08 | *pBR322 Ori kanR T7p-****AmUGT2^F117P^****-T7t* | This study |
| pPE09 | *pBR322 Ori kanR T7p-****AmUGT2^F196C^****-T7t* | This study |
| pPE10 | *pBR322 Ori kanR T7p-****AmUGT2^F196Y^****-T7t* | This study |

**Supplementary Table S3 Assembled DNA constructs used in this study.**

| **ID** | **DNA fragments^a^** |
| --- | --- |
| M1 | *p426GPD backbone us-CYC1t-****PlOMT9****-GAL1p-GAL7p-****AmI3'H****-HIS5t-FBA1t* |
| M2 | *p426GPD backbone us-CYC1t-****PlOMT9****-GAL1p-GAL7p-****MtI3'H****-HIS5t-FBA1t* |
| M3 | *HIS5t-FBA1t-****GmUGT****-GAL10p-p426GPD backbone ds* |
| M4 | *HIS5t-FBA1t-****GuUGT6****-GAL10p-p426GPD backbone ds* |
| M5 | *HIS5t-FBA1t-****AmUGT2****-GAL10p-p426GPD backbone ds* |
| M6 | *ELP3 us-GAL1p-****Ge2HIS****-FBA1t-TPS1t-****GmHID****-GAL10p-ELP3 ds* |
| M7 | *FAS1p us-GAL7p-****Ge2HIS****-pYX212t-IDP1t-****GmHID****-GAL2p-FAS1p ds* |
| M8 | *XII-1 us-PRM5t-****PlOMT9****-SkGAL2p-SuGAL2p-****MtI3'H****-SPO1t-GAT2t* |
| M9 | *SPO1t-GAT2t-****AmUGT2****-SeGAL2p-XII-1 ds* |
| M10 | *XI-1 us-CYC1t-****PlOMT9****-GAL1p-GAL7p-****MtI3'H****-HIS5t-FBA1t* |
| M11 | *HIS5t-FBA1t-****AmUGT2****-GAL10p-XI-1 ds* |
| M12 | *XI-3 us-HSP26t-****PlOMT9****-SkGAL10p-SkGAL1p-****MtI3'H****-IDP1t-TIP1t* |
| M13 | *IDP1t-TIP1t-****AmUGT2****-SmGAL2p-XI-3 ds* |
| M14 | *XII-3 us-CYC1t-****MET6****-tHXT7p-XII-3 ds* |
| M15 | *XII-3 us-CYC1t-****SAM2****-tHXT7p-XII-3 ds* |
| M16 | *XII-3 us-CYC1t-****MET6****-tHXT7p-TDH3p-****SAM2****-HIS5t-XII-3 ds* |
| M17 | *XII-4 us-CYC1t-****MET6****-tHXT7p-TDH3p-****SAM2****-HIS5t-FBA1t* |
| M18 | *HIS5t-FBA1t-****MET13^S443F^****-PGK1p-XII-4 ds* |
| M19 | *XII-3 us-CYC1t-****PGM2****-tHXT7p-TDH3p-****UGP1****-HIS5t-XII-3 ds* |
| M20 | *XII-3 us-CYC1t-****PGM2****-tHXT7p-TDH3p-****UGP1****-HIS5t-FBA1t* |
| M21 | *HIS5t-FBA1t-****URA6****-TEF1p-PGK1p-****YNK1****-IDP1t-XII-3 ds* |
| M22 | *XII-3 us-TDH3p-****GmCHIL****-CYC1t-XII-3 ds* |
| M23 | *XII-3 us-TDH3p-****SmCHIL****-CYC1t-XII-3 ds* |
| M24 | *XII-3 us-TDH3p-****OsCHIL****-CYC1t-XII-3 ds* |
| M25 | *HIS5t-FBA1t-****AmUGT2^L11G^****-GAL10p-p426GPD backbone ds* |
| M26 | *HIS5t-FBA1t-****AmUGT2^F196C^****-GAL10p-p426GPD backbone ds* |

^a^ Bold font indicates genes expressed; *p*, indicates promoter; *t*, indicates terminator; underline, indicates up- stream (*us*) and down-stream (*ds*) sequences of Cas9-targeting locus or backbone of plasmid for homologous recombination.

**Supplementary Table S4 Codon optimized genes used in this study.**

| **Gene (GenBank accession no.)** | **Sequence (5′-3′)** |
| --- | --- |
| *AmOMT*  (KF355972.1) | ATGGGTTCTAGGTACGTCCAAAAAGCTAACGACTTGTTTGAAGGTCAGACTTTGTTGTACGCTCAAATCTTCGGTTACTTGAAGACTGTCTGCTTGAAATGGGCTGTTCAATTGGGTATTCCCGACATCATTAAGAACCACGGCGAGTCTATTACTTTGCCAGAGTTGTTGTCTAAATTGAAGGTCCCACCATCTAAGACTTCTTGCGTTCCAAGATTGATGAGATTCTTGGCCCACAATAGGATTTTCGACATTCACGTCAATCAGAAAGGTCACTTGTCTTACTCTTTGACTCCAGCTTCTGAATTGTTAGTCTCTTCTTCTGACCATTGTTTGTCTCCAGTCGTCACTATGTTTACTAACCAGGTCTTGATGGGTGTCAATCATCATTTGGGTGAATGGGTTTGTGGTGAAGTTCCCACTTTGTTTGAAGTTGCCTTGGGTACTTCTTTTTGGGAATTGGTCAAGGATAAGCCATCTTACATGAACTTGTTCAATGAGGGTATGGCTTCTGATTCTAAGATGGTTGACTTGGCTTTGAAGAACTACTCTTCCATTTTTGAGGGTATCGACTCCATTGTTGATGTTGGTGGTGGTACTGGTACTACTGCTAAGATTATGTCTGCCAAGTTCTCTAACTTGAAGTGCATTGTCTTCGATTTGCCACATGTCGTTGCTAATTTGTTGGGTTCTGACAACTTGTCTTATGTTGGCGGTGACATGTTTATCTCTATTCCAAAGGCTGATGCTGTTTTGTTGAAGTGGATCTTGCATGATTGGACTGACGAGAAGTGTATTGAGATTTTGGAGAAGTGTAAGTACTCTGTCTCTTCTAAGGAGTCTAAAGGCAAGGTCATTATCATTGACACTGTCATCATTGAGAAGGAGGACGATCAGTATATGGCTGACACTAAGTTGTCTATGGACATTTTCATGTTGGGTTTGAAGGGCAAAGAAAGAACTGAGAAGGAGTGGAAACAGTTGTTCATTGAGGCTGGTTTCAAGCACTATAAGATCTTCCCCATTTTCGGCTTCAGGTCTTTGATTGAGGTCTACCCATGA |
| *PlOMT9*  (KP057892.1) | ATGGGTTCTAACAACGGTAGAAAGGCTTCTGAGATTTTCCAAGGTCAGGCTTTGTTGTATAGACACATGTTCGCCTTTGTCGATTCTATGTGCTTGAAGTCTATTGTCGAGTTGGGTATCCCAAACATTATTCACAAACACGGTCAGCCAATTACTTTGTCTGAGTTGATGTCCATTTTGCAAGTCCCACCAGCTAAAGTTGGTCATGTTCAGTCCTTGATGAGATATTTGGCCCACCATGGTTTTTTCGAGAGGTTGAGGATTCATGAAAAGGACGCCTATGCTTTGACTGCTGCTTCTGAGTTGTTGGTTAAAGGTACCGAGCCATATTTGGCTCCAATGGTCGAATGTATGTTGGATCCAACTTTGTCTGCTTCTTTCCATCAGATGAAGAAGTGGGTTTACGAAGAGGACTTGTCTGTCTTTGACATTTCTTTGGGTTCTAACTTGTGGGACTTCTTGACTAAAAACCCAGCCTACAACGAGATTTTCAACGAGGCTATGGCTTCTGATTCTCAGATGTCTAACTTGGCTTTGAGGGATTGTAAGTTGGTTTTCGAAGGCTTGGAGTCTATTGTTGATGTTGGTGGTGGTACTGGTACTACTGCTAAGATTATCTGCGAGGCTTTTCCAAACTTGAAGTGTATCGTCTTCGATAGACCACAGGTCGTTGAAAATTTGTTGGAGAACAACAATTTGACTTACGTTGGTGGTGACATGTTCAAATCTATTCCAAAGGCCAACGCTGTTTTGTTGAAGTGGATTTTGCACGATTGGACTGACAAAGACTGTAGGAAGATTTTGGAGAACTGCAAAGAGGCCATTTCTAACAATTCTAAGCGCGGTAAGATTATCATTATCGACATCGTCATTAATGAGAAGCAGGACGAGCATAAGGTTACTGAGTTGAAGTTGTTGTTGGACGTTGCTATGGCTTGTGTTTTGAACGGTAAGGAAAGAAACGAAGAGGAGTGGAAAAAGTTGTTCATGGAGGTCGGTTTGCAAGACTACAAGATTTCTCCATTGACCGGTTACTTGTCTTTGATTGAGATCTACCCCTAA |
| *MsOMT8*  (U97125.1) | ATGGCCTCTTCTATTAACGGTAGGAAACCATCCGAGATTTTCAAAGCTCAGGCTTTGTTGTACAAGCACATTTATGCCTTCATCGACTCTATGTCTTTGAAATGGGCTGTCGAAATGAACATCCCAAACATTATTCAGAACCACGGTAAGCCAATTTCCTTGTCTAACTTAGTCTCTATTTTGCAGGTCCCCTCTTCTAAGATTGGTAACGTCAGGAGATTGATGAGATACTTGGCTCACAATGGTTTCTTCGAGATTATTACTAAGGAGGAGGAGTCTTACGCTTTGACTGTTGCTTCTGAATTGTTGGTTAGGGGTTCTGATTTGTGTTTGGCTCCAATGGTTGAATGTGTTTTGGACCCAACTTTGTCTGGTTCTTATCACGAGTTGAAGAAGTGGATTTACGAGGAAGACTTGACTTTGTTTGGTGTCACTTTGGGTTCTGGTTTTTGGGACTTCTTGGATAAGAACCCCGAGTACAACACTTCTTTTAACGACGCTATGGCTTCTGACTCTAAGTTGATTAACTTGGCTTTGAGGGATTGCGATTTCGTCTTTGATGGCTTGGAGTCCATTGTTGATGTTGGTGGTGGTACTGGTACTACTGCTAAGATTATTTGCGAGACTTTCCCAAAGTTGAAGTGCATTGTTTTCGACAGGCCACAAGTTGTTGAAAATTTGTCCGGCTCTAACAACTTGACTTATGTTGGTGGCGACATGTTTACTTCTATTCCAAACGCTGACGCTGTTTTGTTGAAGTACATTTTGCATAACTGGACCGATAAAGACTGCTTGCGCATTTTGAAAAAGTGCAAGGAGGCTGTCACTAACGATGGTAAAAGGGGTAAGGTCACTATTATCGACATGGTCATTGACAAGAAGAAGGACGAAAATCAGGTCACTCAGATCAAGTTGTTGATGGACGTTAACATGGCTTGTTTGAATGGTAAGGAGAGGAATGAGGAAGAGTGGAAGAAGTTGTTTATTGAGGCTGGTTTCCAGCACTATAAGATTTCTCCCTTGACTGGTTTCTTGTCTTTGATCGAGATCTACCCATGA |
| *GmOMT2*  (XM_006586386.3) | ATGGCCTCTTCTTTGAACAACGGTAGAAAGGCTTCTGAGATTTTCCAAGGTCAAGCCTTGTTGTACAAGCACTTGTTGGGCTTTATTGACTCTAAGTGCTTGAAGTGGATGGTCGAGTTGGATATCCCAGACATCATTCATTCTCATTCTCACGGTCAGCCAATTACTTTTTCTGAGTTAGTCTCCATTTTGCAAGTCCCCCCAACTAAAACTAGACAAGTCCAGTCTTTGATGAGGTATTTGGCTCACAACGGTTTCTTCGAGATTGTTCGCATCCATGATAACATTGAGGCTTACGCTTTGACTGCTGCTTCTGAATTGTTGGTCAAGTCTTCTGAGTTGTCTTTGGCTCCAATGGTTGAGTATTTCTTGGAGCCAAATTGTCAAGGTGCTTGGAATCAATTGAAAAGGTGGGTTCATGAGGAAGACTTGACTGTTTTTGAGGTATCTTTGGGTACTCCATTCTGGGACTTCATTAATAAGGACCCCGCTTACAACAAGTCTTTTAACGAGGCTATGGCTTGTGATTCTCAGATGTTGAATTTGGCTTTCAGGGACTGTAATTGGGTTTTCGAGGGTTTGGAGTCTATTGTTGATGTTGGTGGTGGTACTGGTATTACTGCTAAGATTATCTGCGAAGCTTTCCCAAAGTTGAAATGCATGGTTTTGGAAAGACCAAATGTCGTTGAAAACTTGTCTGGCTCTAACAACTTGACTTTTGTCGGTGGCGACATGTTTAAATGCATTCCCAAGGCTGATGCTGTTTTGTTGAAGTTGGTCTTGCACAATTGGAATGACAACGACTGCATGAAGATTTTGGAGAACTGCAAGGAGGCTATTTCTGGTGAATCTAAGACTGGTAAGGTCGTCGTTATTGACACTGTCATCAACGAAAACAAAGACGAGAGACAGGTTACTGAGTTGAAGTTGTTGATGGACGTTCATATGGCCTGCATCATTAATGGTAAGGAGAGGAAGGAAGAAGATTGGAAGAAGTTGTTCATGGAGGCTGGTTTTCAGTCTTACAAGATCTCTCCATTCACTGGCTACTTGTCTTTGATTGAGATCTACCCCTGA |
| *AmI3′H*  (JQ609280.1) | ATGGCTCCCTTGTTGTATTACTCTTTGTTGTCCTTGGCCTTCATTTTGACCGTCAAGATTATCTTGCAGATTCAGTCCAGGAGGTTGAAAAACTTGCCACCAGGTCCACCAACTATTCCAATTATTGGCAACTTGCACCACTTGAAACATCCATTGCACAGGACTTTTACCACTTTGTCTCAAAAGTACGGCGACATTATTTCTTTGTGGTTCGGTTCTAGATTGGTCGTTGTTGTTGCTTCTCCATCTATTGCTCAGGAGTGCTTCACTAAAAATGACGTCGTCTTGGCTAACAGACCAAAATTCTTGACTGGTAAGTACATCTTCTATAACTACACCACCTTGGGTTCTGCTTCTTATGGTGATCACTGGAGGAATTTGAGGCGCATTACTACTATTGACGTCTTGTCTAACCATCGCTTGAACTCTTTTCACGGTGTTAGAAAGGACGAAACTTTGAGATTGGTCGAGAAGTTGAGGAATGACGTCGTTAAGGAAGGTAACTTCTCTTTCACTGACGTCGAGTTGAGACATAGGTTGACTGAGATGACTTTCAACGCTATGATGAGGATGATTTCTGGCAAGAGATACTACGGTGACGATGGTGATGTCACTGATGTTAAGGAAGCTAAGCAATTCCGCGACATTATTTCCGAGATTTTGTCTTTGTTGGGCGCTAACAATAAGGGTGACTTCTTGCCATTGTTGAGGTTGTTTGACTTGGATCACTTGGAGAAGAGATGCAAGAGAATCTCTAAGAGAGCTGACGCTTTTTTGCAAGGTTTGATTGAGCAACATCAGAACGCTAACCATTCTGATAACGACGGCGATACTATGATTGACCACTTGTTGAAGTTGAGAGAAACTCAGCCCGAGTATTATTCTGACCACATGATTAAGGGTTTGATTCAGGCTATGTTGTTGGCTGGTACTGATACTTCTGCTGTCACTATTGAATGGGTTATGGCTGAGTTGTTGAATAATCCAGAGGTCTTGAAGAAAGCTAAGAAGGAGATCGAAACCACTATTGGTAAGGAGAGGTTGGTTGAAGAACAGGACTTGTCTCAATTGCCATACTTGCAGAACGTCATTTCTGAGGCTTTGAGATTGCATCCACCAGCTCCATTGTTGTTGCCACATTCTGCTTCTGAAGACTGTACTATTGGTGGTTTCAATGTCCCAAAGGACACCATTATTTTGACTAACATTTGGGCCATTCACAGAGATCCAGAATTGTGGACTGATCCATCTTCTTTCAAACCAGAGAGGTTCGAAAAAGAAGGTGAGGTCAACAAGTTGTTGTCTTTCGGTTTGGGTAGAAGAGCTTGTCCAGGTTTGTCTTTGGCTCAAAGAACTGTCGGTTATACTGTCGGTTTGTTGATTCAAGGTTTCGAATGGAAGACTGAGTCTGAAGAGAAGTTGGATTTGGCTGAGGGTAAAGGTATTACTATGCCAATGAAGTTTCCATTGCGCGCTATGTGTAAACCATTGCCAATTGTCAATGACATTATGAAATGA |
| *MtI3′H*  (AY278228.1) | ATGGCTTTGTTCTACTACTCCTTGTTGTCTTTGTCTTTCATCATTACTATCAAGATCTTGTTGAAGATCACTTCTAGGCGCTTGAAAAACTTGCCACCAGGTCCACCAACTATTCCAATTATCGGTAACTTGCACCACTTGAAACACCCATTGCACAGAACTTTCACCACTTTGTCTCAAACTTACGGCGACATTTTCTCTTTGTGGTTCGGTTCTAGGTTGGTTGTTGTTGTTTCCTCTCCATCTTTGGCTCATGAGTGCTTTACTAAGAACGACATTATTTTGGCTAACCGCCCAAGATTTTTGACTGGCAAGTACATTTTCTACAACTATACCACTTTGGGCTCTGCTTCTTATGGTGATCATTGGAGGAATTTGAGGAGGATTACCACTATCGATGTCTTGTCTAACAACAGGTTGAACTCTTTCTTGGGTGTTAGAAGGGACGAAACCAATAGGTTGATCCAGAAGTTGTTGAAGGACGTTGTCTCTGAAGGTTTTGGTTTCACTAAGGTTGAGTTGAGGCCAAGATTGACTGAGATGACCTTCAATGCTATGATGAGGATGATTTCTGGCAAGAGGTACTATGGTGACGATGGTGATGTTTCTGATGTTGAAGAAGCTAAGCAGTTCCGCGAAATTATTTCCGAGATGATGTCTTTGTTGGGCGCTAATAACAAAGGTGACTTCTTGCCATTGTTGAGGGTCGTTGATTTGGACAACTTGGAAAAGAGGTGTAAGAGGATTGCTAAAAGGTCTAACGCTTTCTTGGAGGGTTTGATTGAAGAACACAGGAGGGGTAACATTCATTCTGACGGCGGTACTATGATTGACCACTTGTTGAAGTTGTCTGAATCTCAGCCAGAGTATTATTCTGACCACTTGATCAAGGGTTTGATCCAGGGTATGTTGTTGGCTGGTACTGATACTTCTGCTGTCACTATTGAATGGGTCATGTCTGAATTGTTGAATCACCCAGAGGTCTTGAAAAAGGCTAAGGAGGAATTGGACACTCAGATTGGTAAGAACAAGTTGGTTGATGAGCAGGATTTGTCTAAGTTGCCATACTTGCAGAACATTATTTCTGAGACTTTGAGATTGCATCCACCAGCTCCATTGTTGTTGCCACACTATTCTTCTGAAGACTGCACTATCGGTGAATTTAACGTCCCAAAGGACACTATCATTTTGACTAACGTCTGGGGTATTCACAGAGATCCAAAGCATTGGAATGACGCCTTGTCTTTTAAGCCAGAGAGATTCGAAAAAGAGGAGGAGGTCAATAAAGTCATGGCTTTCGGTTTGGGTAGAAGAGCTTGTCCAGGTTTGTCTTTGGCTCAAAGGACTGTTGGTTTTACTGTCGGTTTGTTGATTCAGTGTTTTGAGTGGGAAAGAGAGTCTGAAGAGAAGTTGGATATGATGGAGGGTAAGGGTATTACTATGCCCATGAAGATTCCATTGAGGGCTATGTGTAAAGCTTTGCCAATTGCTAACGACGTCACCAAATGA |
| *ThF3′H*  (AB057672) | ATGTCTCCATTGGCTTTGATGATTTTGTCTACCTTGTTGGGCTTTTTGTTGTATCACTCTTTGCGCTTGTTGTTGTTTTCTGGTCAGGGTAGAAGATTGTTGCCACCAGGTCCAAGACCATGGCCATTGGTTGGTAATTTGCCACATTTGGGTCCAAAACCACATGCTTCTATGGCTGAATTGGCTAGAGCTTATGGTCCATTGATGCACTTGAAAATGGGTTTTGTCCACGTTGTCGTTGCTTCTTCTGCTTCTGCTGCTGAACAATGTTTGAGAGTCCATGACGCTAATTTCTTGTCTAGGCCACCAAATTCTGGTGCTAAACACGTCGCTTACAATTATGAGGACTTGGTCTTCAGACCATATGGTCCAAAATGGAGGTTGTTGAGGAAAATTTGCGCTCAGCACATCTTTTCTGTTAAGGCTATGGACGATTTTAGAAGAGTCAGGGAAGAAGAAGTCGCTATTTTGTCTAGAGCTTTGGCTGGTAAAAGAGCTGTTCCAATTGGTCAGATGTTGAACGTTTGTGCTACTAACGCTTTGTCTAGAGTTATGATGGGCAGAAGAGTTGTTGGTCATGCTGATGGTACTAATGATGCTAAAGCTGAGGAGTTTAAAGCTATGGTCGTCGAATTGATGGTTTTGTCTGGCGTTTTCAACATCTCTGACTTCATCCCATTTTTGGAGCCCTTGGATTTGCAAGGTGTCGCTTCCAAGATGAAAAAATTGCACGCTAGGTTTGATGCTTTCTTGACTGAGATTGTCAGGGAAAGATGTCACGGCCAAATTAATAATGGTGGTGCCCACCAAGATGATTTGTTGTCCACTTTGATCTCTTTCAAGGGTTTGGATGACGGTGATGGTTCTAGATTGACTGACACCGAAATTAAGGCTTTGTTGTTGAACTTGTTTGCTGCTGGTACTGATACTACTTCCTCTACTGTTGAATGGGCTGTTGCTGAATTGTTGAGGCATCCAAAAACTTTGGCTCAAGTCAGACAAGAATTGGATTCCGTCGTTGGTAAAAACAGGTTGGTATCTGAAACTGACTTGAATCAGTTGCCATACTTGCAAGCTGTTGTCAAAGAGACTTTCAGATTGCATCCACCAACTCCATTGTCTTTGCCAAGATTGGCTGAAGACGACTGTGAAATTGATGGCTACTTGATTCCAAAGGGTTCCACTTTGTTGGTTAACGTTTGGGCTATTGCTAGAGATCCAAAAGTTTGGGCTGATCCATTGGAATTTAGGCCAGAGAGATTTTTGACTGGTGGTGAAAAAGCTGACGTTGACGTCAAAGGTAATGACTTCGAGTTGATTCCATTTGGTGCCGGTAGAAGAATTTGTGCTGGTGTTGGTTTGGGTATTAGAATGGTCCAGTTGTTGACTGCTTCTTTGATTCACGCTTTCGATTTGGATTTGGCCAATGGTTTGTTGCCCCAAAACTTGAATATGGAAGAGGCTTACGGTTTGACTTTGCAAAGAGCTGAACCATTGTTGGTTCATCCAAGATTGAGATTGGCTACTCATGTCTACTGA |
| *GmUGT*  (AB904893.1) | ATGACCATGAAGGACTCTATCGTTTTGTATTCCGCTTTGGGTAGAGGTCATTTGGTTTCTATGGTCGAATTGGGCAAGTTGATTTTGTCTCACCACCCATCTTTGTCCATCACCATTATCTTCTTGACTCCCCCACCAAATCAAGATACTCCAACTTCTCCAACTGCTTTTACTTGTGATGCTACCGCTAAGTATATTGCTGCTGTAACTGCTTCTACTCCATCTATTACTTTCCACCGCATCCCACAAATTTCTGTCCCAACTGTTTTGCCACCAATGGCTTTGACTTTTGAATTGTGCAGAGCTACTGGTCATCACTTGAGGAGGATCTTGAACTCTATTTCTCAGACTTCTAACTTGAAGGCCATTGTTTTGGACTTCATGAACTACTCTGCTGCTAGGGTTACTAATGCTTTGCAGATTCCAACTTACTTCTACTACACTTCTGGTGCTTCTACTTTGGCTATTTTCTTGCAGCAGATTATTATCCACGAGAATTCCACCAAGTCTTTCAAGGACTTGAACATGCACTTGGTTATTCCAGGTTTGCCAAAGATTCATACTGACGACTTGCCAGAACAAATGCAAGACAGAGCTAATGAGGGTTACCAGGTCTTTATTGACATTGCCACTTGCATGAGGGATTCTGACGGTGTCATTGTTAATACTTGCGAGGCTATGGAAGGTAGAGTTGTTGAAGCTTTCTCTGAAGGTTTGATGGAAGGTACTACTCCAAAGGTTTTCTGTATTGGCCCAGTCATTTCTTCTGCTCCATGCAGAAAAGATGACAACGGTTGTTTGTCTTGGTTGGATTCTCAACCATCTCACTCTGTTGTTTTCTTGTCTTTTGGTTCTATGGGTCGCTTTTCTAGAACTCAGTTGAGGGAGATTGCTATTGGTTTGGAGAAATCCGAACAGAGGTTTTTGTGGGTTGTCAGATCTGAGTTTGAGGAAGGTGATTCTGGTGAACCACCATCTTTGGATGAATTGTTGCCCGAAGGTTTTTTGGAGAGGACTAAGGAGAAAGGTTTGGTCGTTAGAGATTGGGCTCCACAAGCTGCTATTTTGTCTCATGATTCCGTCGGTGGTTTTGTTACTCATTGCGGCTGGAATTCTGTTTTGGAGGCTGTTTGTGAAGGTGTTCCAATGGTTGCTTGGCCATTGTATGCTGAACAGAAGTTGAACAAGGTCATTTTGGTCGAGGAGATGAAAGTTGGTTTGGCTGTCAAGCAGAATAAGGATGGTTTAGTCTCTTCTACTGAGTTGGGTGATAGAGTTATGGAGTTGATGGACTCTGACAAGGGTAAAGAAATTAGGCAGCGCATTTTCAAGATGAAGATCTCTGCTACTGAGGCTATGGCTAAAGGTGGTTCTTCTATCATGGCTTTGAACAAGTTGGTTGAGTTGTGGAGAGAACACTGA |
| *GuUGT6*  (MK534517.1) | ATGAAAGACACTATCATCTTGTATCCAGCTTTGGGTTCTGGTCATTTGATGTCTATGGTCGAATTGGGTAAGTTGATCTTAACGCAGAATCCATCTTTGTCCATCACCATCTTGATCTTGACTCCACCAAATACCAAGATCAACAACAACAATAACAACAACACTTTCGGTTGTGAAGATTTCCCATCCATCACTTTCCACTACATTCCACCAATTTCTTTCCCAAACACTTTGCCACCACATATTCTGACTCTGGAATTGTGTAGACTGTCTAATCACCATGTTCATCATGTCTTGCAGTCTATCTCCAAGACTTCCAACTTGAAGGCAATTGTTCTGGATTTCTTGAACTACTCCACTACTCACATCACCTCTACTATCGATACTCCAACTTACTTCTACTACACCTCTGGTGCTTCCACTTTGTCTGTATTCTTGCAGTTGCCAACCATTCACAAGAAGTCTAACCGTAGCTTGAAAGAGGACTCTCACATGCACTTGAGAATACCAGGTTTGCCAGCTATTCCAGTTGCTGATATGCCAGAAGAAGTCAAAGACAGAGAATCTCAGTCTTACCAAGTTTACTTGGAGATTGCAACCTCTATGAGAGATTCTGATGGTGTCATCATCAACACCTTCGATGGTATCGAAGGTAGAGCTGTTAGAGCTTTGTCTGCTGGTTTGTGTTTGCCAGAGGGTAATACTCCACCAGTATTCTGCATTGGTCCAGTTGTTTCTGGTTCTGCTAAGACTACTGGTAGAGATGATGAGAACGGTTGTTTGTCTTGGTTGGATTCTCAACCATCAAGATCCGTTGTTCTGTTGTCTTTCGGTTCTATGGGTAGATTCTCTCGTTCACAATTGAGAGAAATTGCTGTTGGTTTGGAAAGATCAGGTCAAAGATTCTTGTGGGTCGTTAGATCCGAATTAGGTGGTGGTGTTGATTCTGGTGATGAACCATCTTTGGAAGAGTTGTTGCCAGAAGGTTTGTTGGAAAAAACCGAAGGTAAAGGTTTGGTTGTCAGAAACTGGGCTCCACAAGCTGCTATTCTAAACCATGATTCTGTTGGTGGTTTCGTTACTCATTGTGGTTGGAACTCTGTTCTTGAAGCTATCATCTGTGGTGTTCCAATGGTTGCTTGGCCATTGTATGCTGAACAGAAGTTGAACAAGGTTATTCTGGTCAAAGAGATGAAAGTTGCTTTGGAATTGAACAACGAATCCAATGGCTTCGTTTCTGGTACTGAATTGGGTGAAAGAGTTAAAGAATTGATGGAGTCTGAAAGAGGCAAAGAAATCAGAGAAAGAGTCTCCAAGATGAAGGTTTCTGCTAAAGAAGCTAGAGGTGGTGGAGGTTCTTCTTTGGTTGCTTTGAAGAAACTAGCTGAATCTTGGAAAGAGCATTCTTGTCTGAATAACAACCTGTCTCCAAACTCTCCATTCAACTTGCATTGGCAATGA |
| *ApUGT3*  (MH379335.1) | ATGGGTTACCACTCTCATATTGGTGTTTTGGCTTTCCCATTTGGTTCTCATGCTGCTCCATTGTTGGCTTTGGTTAGACAATTGGCTGAATCTTCTCCAGGTACTTGCTTTTCTTTCTTCAACACCGCTGATTGCAACAGAGCTATTTTGGCTGATCACGTATCTCCAAACATTAAAGCTTACGACGTCAGAGATGGTGCTCCAGAAGGTGCTGCTGCTGCTGCTTCTCATATGGAAGCTATTAGGTTGTTTTTGGCTGCTTCCCCAGGTAATTTTGAAGCTGGTATGGAAGAAGCTGAAAGAGCTGCTGGTGTTGGTATTGGTTGCTTGATTACCGATGCTTTCTTGTGGTTTGCTGCTGATTTGGCTGCTAATAGAAGAATTCCATGGGTTGCTTTGTGGACTGCTGCTGCTTGTGCTTTGGCTACTCATATGTACACTCAGGAGATTGTTAAGGCTGTTGCTTCTCCAGCTGAAGCTACTGGTGAATCTATTCAGCAAGTCTTGTCTTTTACTCCACCAGGTTTGCCACCATTGCAAATTGCTGATTTGCAGCCAGAGATTTTTATTGACGACAGGAATCCATCTCCATTGGCTGCTACTATTAACAACATGGTCGAGAAGTTGCCAAAATCTACTGCTGTCGTCTTGAATTCTTTCGAGGAGATCGATCCAATTGTCGCTCAAGACTTGAAATCTAAGTTCAACCACTTCTTGTCTGTTGGTCCAGCTGCTTTGAGAAGATTGCCACAACCACCACCAGGTGATGATTCTGGTTGTTTGTCTTGGTTGGAGAAATTGCCAACTCCAAGGTCTGCTATTTACGTTTCTTTCGGTACCGTTATTGTTCCACCAGAGAATGAATTGTTGGCTTTGGCTGAAGCTTTGGAATTTTGCAAGTTCCCATTCTTGTGGTCTTTGAAAGAAGCTGCTGCTAAAGCTTTGCCAGAAGGTTTTTTAAGACGCACAGCTGAATATGGTAGAGTTGTTCCATGGGCTCCACAACCATGGATTTTGAGACATGGTTCCGTCGGTTTGTTTGTTACTCATGGTGGCTGGAATTCTATCTTGGAGTCTATTTGCGGTGGTGTTCCAATGGTTTGTAGACCATTCTTTGGTGACCAGAAGTTGAATGGTAAGATGGTTGAAGACTCTTGGAAGATTGGTGTTAGAGTTCGCGATGGTGTTTTTAGGAAGGATGAAACTGTTAGGGTCTTGCAAAGAATGATGTCCTCTAGAGAAGGTGATGACGCTAGAGAGAACGTTGTCAAGATGAAAGAGAAGGCTGAAAAAGCTGTCGGTAATGATGGTTCTTCTACCAAGAACTTCAAGAAGTTGTTGGAGATCATCGGTATCTCTAAGTGA |
| *AmUGT1*  (KF355973.1) | ATGGAGTCTAAGACCGACTCTATTAAGATGTTCTTTTTCCCATTCGTCGGTGGTGGTCATCAAATTCCAATGATCGATACTGCTAGAGTCTTTGCTGCTCATGGTGCTATGTCTACTATTTTGACCACTCCATCCAACGCTTTGCATTTCCAGAAGTCTATTACTAGGGACCAACAGTCTTGTTTGCCAATTACCATCCACTTGTTGACTACCACTGTCGACATTACTGATACTGACATGTCTGCTGGTCCAATGATTGACACCTCTATTTTGTTGGAGCCCTTGAGAGAATTCTTGTTGCAACATCCCCCAGATTGTATTGTTGTCGACATGTTTCATAGGTGGGCTAACGATGTCATTGACGAGTTGAAGATTCCACGCATTTTCTTCACTGGCAACGGTTGTTTTCCAAGATGCGTCCACGAGAATATTTCTAGGCATGCCGTCTTGGATAACTTGTCTTCTGACTCCGAGCCATTTATTGTTCCAGGTTTGCCAGACAAAATTGAGATGACTAGGTCACAGTTGCCAATTTTCGCTAGGAACCCATCTCAATTTCCAGATAGGTTGAGACAGATGGAAGGTAAATCTTTCGGTACTGTCATCAACTCTTTCTATGACTTGGAGCCAGCTTATGCTGACTACATTATTAACGTCTTGGGTAAGAAAGCTTGGTTGGTTGGTCCAGTTTCTTTGTGCAACATTTCTGTCAAGGACAAGACTGAAAGAGGTAAGCAGCCAACTGTTGATGAACAGTCTTGTTTGAACTGGTTGAACTCCAAGAAACCAAACTCTGTCATCTACGTATCTTTCGGTTCTTTGGCTAGATTGCCACCAGAACAGTTGTGTGAAATTGCTTACGGTTTGGAAGCTTCTGAGCAGTCTTTTATTTGGGTCGTCGGTAAGGTTTTGAACTCTTCCAAGAAGGAGGAAGTTGGTGGTGATCAAAATTGGTTGCCAAATGGTTTTGAGGAAAGGATGAAGGAGACTAACAAGGGCTTGATTTTGAGAGGTTGGGCTCCACAATTGTTGATTTTGGAGCATGCTGCTGTTGGTGGTTTTGTTACTCATTGCGGCTGGAATTCTACTTTGGAAGGTGTTTGTGCTGGTGTTCCAATGGTTACTTGGCCATTGACTGCTGAACAATTCTCCAACGAGAAATTGATTACCAACGTCTTGGGTATTGGTGTTCAAGTTGGTTCTAGAGAATGGTGGTCTTGGAATGGTGAATGGAAACAAGTTGTTGGTCGCGAAAGAGTTGAATTGGCTGTCAAGAAGTTGATGACTAGATCTGAAGAGGCTGAAGAAATGAGAAGGCGCGTCAAACATATTGCTGGTAATGCTAGGAGGGCTATTAAAGAAGGTGGTACCTCTTACGCTGATATCGACGCTTTGATTCAGGAGTTGAAAGCCAATAGGTTCACTTCTCAGGTTTGA |
| *AmUGT2*  (OQ876891.1) | ATGAAAGACACTTTGGTTCTATATCCAGCTTTGGGTAAAGGTCATCTAAACTCTATGATCGAGTTGGCTAAATTGATCTTGACTCAGAATCCATCTTTCTCTATCACTATCTTGATCTTAACACCACCAAACACTACCAATAACACCTCTTCCACTAACACTACTACCTTTGGTTGTGAATCTTTGCCATCTATTACCTTTCACCATATTCCACCAATCAATCTGCCAACTACTTTGCCACCACAAGTCTTGCCATTTGAACTATGTCATGTTTCTAACAACCATGTTCAACATGTCTTGAGATCCATCTCTAAGACCTCTAATTTGAAGGGTGTCATCTTGGACTTCTTTAACTATTCTACTACTCAAATTACCAACAATTTGGAGATTCCAACCTACTTCTACTACACCTCTGGTGCTTCTACACTTGCCATTATGTTGAAGATGCCAACTATCCATCAAATCACCACTACTTCATTCAAAGACAACTTGAATATGCATTTGAGAATACCAGGTTTGCCAAAGATTCCATTGGTTGATATGCCAGAACACACTAAAGACAGAGAATCCAAATCTTACAGAGTCTTTCTTGATATTGCTACTTGTATGAAAGATTCTGATGGTATTATCATTAACACCTTTGAGGCTATTGAGAAGAGAATCATTGAAGCTATGAACAAGGGTTTGGTCTTGTCTGAAGGTACAACTCCACCACTGTTCTGTATCGGTCCAATGGTTTCCAATTCCACTACTTGTTGTGAGAAAGACGAAAGAGGTTGTTTGTCTTGGTTGAACTCTCAACCATCAAGATCCGTCGTCTTGTTGTCATTTGGCTCTTTGGGTACTTTCTCCAGAACTCAGTTGAAAGAAATTGCCATTGGTCTTGAGAAATCTGGTCAAAGATTCTTGTGGGTTGTTAGGTCTAAGAACGAATTGGTTGAAAGAGTTGATTCTGAAGAACTATCACTAGATGAACTGTTGCCAGAAGGCTTTCTGGAAAGAACCAAAGACAAAGGTATGGTTGTCAGAAATTGGGCTCCACAAGCTGCTATTCTGTCTCATGATTCTGTTGGTGGTTTCGTTACTCATTGTGGTTGGAATAGTGTGTTGGAAGCTGTTACATTTGGTGTTCCAATGGTTGCATGGCCATTGTACGCTGAACAGAAGTTGAACCGTATGATCATGGTTGAAGAAATGAAGGTTGCATTGAAACTGAATGAATTGGAAGATGGTTTCGTATCTGGTACTGAATTGGGTGATAGAGTTAAAGAATTGATGGAGTCTGAATCTGGCAAAGAAATGAGAGAGAGAATCTACAAGATGAAGGTTTCTGCTAAAGAAGCTAAAGGTGGTGGTGGTTCTAGTTTGGTTGACTTGAAGAGATTGCAAGACTCTTGGAGAGATCATGCATCTATGCATTCTTTGTCACCAAATTCTCCATTCTTGTTTAGATAG |
| *AmUGT3*  (OQ876892.1) | ATGAAGGACACTATTGTCTTGTATCCAGGTATGGGTTCTGGTCATTTGATGTCTATGGTTGAATTGGGTAAATTGATTATCACTAGGCACCCATCCTTCCACATTACTATTTTGATTCTGACTCCCCCAAAGATTAACAACAACACTCCAACTCCAAAGAACGATTCTATTACCCAGTATATTGCTTTCGTCAGTTCCACTTTCCCATCTATTACTTTCTTGTACATTCCCACTACTCAGAACTCTTTCCCCACTTCTTTGCCAATGCATTTGTTGACTTTGGAGTTGTCTCCAAGAAATAACCACCACGTCCAGCATTTGTTGCAATCTATTTCTAAGACTTCTAACTTGAAGGCCGTCGTCTTGGACTTCTTGACTTATTCTGCTTCTAAAATTACCACTATCTTGGAGATTCCCACTTACTTCTACTACACCTCTTCTGCCTCTTTGTTGGCTTTGTTCTTGCATTTTCCAACTTTCCACCAAAACGCTAAGAAGCCAATTAAGGAGATTCACATGCACACTGCTATTCAGATTCCAGGCTTGCCAATTATGTCTTTGGAGGATTATCCAGACGATGCTAAAGATCCAACTTCTCAATCTTATAGGGTCTTGTTGGATTCCGCTAAGATTGTCAGAGAGTCTGTTGGTATTATTGTCAATACCTTCGACGACATTGAGAGGAGAGCTATTAAAGCTTTGAAGGAGGGTTTGTGCATTCCAGATGGTATTATTCCACAACTGTTCTTCATTGGTCCCGTCGTTTCTAATTCTACTTCTTGCGAAAAAGACGAGAACGAGTGCTTGTCTTGGTTGAACTCTCAGCCATCTCAGTCTGTCGTTTTGTTGTCTTTTGGTTCTATGGGCTCTTTCTCTTGGACTCAGTTGAAAGAAATTGCTATTGGTTTGGAGAAGTCCGGCCAGAGGTTTTTGTGGGTTGTTAGATGTAAAAACGAGCTGGTCGAGAGGGTTGACTCTGATGGTGGTGTTGAAAGAGTTGATTCTGAAAAATTGTCTCTGGACGAGTTGTTGCCAAAAGGTTTTTTGGAAAGGACTAAGGACAAAGGTATGGTCGTCAGGAATTGGGCTCCACAAGCTGCTATTTTGTCTCATGATTCTGTTGGTGGTTTTGTCACTCACTGTGGTTGGAATTCTGTTTTGGAAGCTGTTACTTATGGTGTCCCAATGGTTACTTGGCCATTGTATGCTGAACAAAAATTGAACAGGGTTATTTTGGTCAAGGAGATGAAGGTTGCTTTGGAGTTGAATGAGTTGGAGAACGGTTTTGTCTCTGCTACTGAATTGGGTGAGAGAGTTAAAGAATTGATGGAGTTGGAATCTGGCAAGGAGATTAGGGAGAGGATTTATAAGATGAAGGTATCTGCTAAAGAGGCTAGGGGTGGTGGTGGTTCTTCTTTGGTTGATTTGAAAAGATTGCAGGACTCCTGGAGGCAACAATAA |
| *AmUGT4*  (OQ876893.1) | ATGGCTGTCAACGGTATGAAGAAGAATAAGGTCGAGTTGATTTTCATTCCCACTCCAGCTATTGGCCATTTGAGATCTGCTTTGGAATTTGCTAAATTGCTGATTAACAACGACAACAACTTGCACATTACTGTCTTGTGCATGAAGTTGCCATTCACTGCTTTTTCTGACTCTTACATTAAGTCCGTCGTCGCTTCTCAGCCACAAATTAAGTTGATTGATCTGCCCGAGATTGAGTCTCCCCCATTGGAAGAATTTTTGAAGTGCCCAGAACATTACATTTTGACTTTCATGGAGTCTTTGATTCCCCACGTCAAGGCTACTATTCAGACTATTTTGTCTTCTTCCTCTATCCCAGTCGTCGGTTTGTTGTTGGATTTTTTCTGTATTACTATGATTGACGTCGGTGACGAGTTCGGTATTCCATCTTATTTGTTTCTGACTTCCAACGTCGCTTTTTTGGGTTTGATGTTCTCTTTGTTGAAGCGCCAGATTGAGGACGTCTTCAACGACTCTGAACCAGATTTGTTGATTCCCGGTTTGTCTAATTTGGTGCCAACTTCTGTTCTGCCAGACCCATGTTTTTCTAAGATTGGTGGTTACTTGGCCTATTACAAGTTGGCCGAAAGATTCCGCGATACTAAGGGTATTATTGTCAACACTATTTCTGAGCTGGAGCAGAAGGCTATTGATGCTTTGTCTGAGGAAAGGACTCCACCAATTTATGCTGTTGGTCCATTGTTGGATTTGAAGTGGTCACCAAATCCAAATTTGGACCAAGTTCAGCACGATTTGATTCTGAAGTGGTTGGATCAACAGCCAGACTCTTCTGTTGTTTTTTTGTGCTTTGGCTCTGCTGGCAGATTTGGTCCATCTCAAACTAGAGAAATTGCTAGGGCTTTGCAGTGTTGTGGTGTTAAATTTTTGTGGACTATGAGGTCACCACCAACTATTGATAATGAGGAAAGAGCTTTGCCAGAGGGTTTTTTGGAATGGATGGATGGTAAAGGTATGATCTGCGAATGGGCTCCACAAGTTGAAGTTTTGGCTCATAAAGCTATTGGCGGTTTTGTTTCTCACTGCGGTTGGAATTCTATTTTGGAATCTTTGTGGTTTGGTGTCCCCATTTTGACTTGGCCAATTTATGCTGAACAACAGTTGAACGCTTTCTGGATGGTCAAAGAATTGGGTTTGGGTATGGAATTGAGGTTGAATTATAGGAAGGGTTCCGACTTGGTCGTCGCTGAAGAAATTGAAAAAGGTTTGAAGCAGTTGATGGAGGAGAGAAACTCTAAGGTTCATAAGAAGGTCCAAGAGATTAAGGAGTTGGCTAGAAAAGCTGTCTTGGATGGTGGTTCTTCTTCTATTTCTATTAGAAAGTTGATTGACAATATTATTGGTAGGAACTGA |
| *GmCHIL*  (NM_001249853.2) | ATGGCTACTGAAGAGGTCTTGGTCGATGAAATCACTTACCCAACCAAGATCACCACTACCAAGCCATTGTCTTTGTTGGGTCACGGTATTACTGACATGGAAATTCACTTTATCCACGTTAAATTCTACTCTATTGGTGTTTATTTGGAACCAGAAGTTGTTGGCCATTTGGACCAATTCAAGGGTAAGTCAGCCAAGGAATTGGAAGACAACGAAGAATTTTTCAACGCTTTGATTTCCGCTCCTGTTGAAAAGTTCATCAGATTAGTTGTCATCAAGGAAATCAAGGGTGCTCAATACGGTGTCCAAATCGAAACTGCTGTTCGTGACAGATTAGCTGCTGAAGATAAATACGAAGAAGAAGAAGAAGAAGCCCTAGAAAAGGTTATTGAATTCTTCCAATCCAAGTACTTCAAGAAGTTGTCCGTCATTACCTACCACTTCCCAGCTAACTCTGCTACCGCTGAAATTGTCGTTTCTTTGGAAGGTAAGGAAGATTCTAAGTACGTTATTGAAAACGCCAATGTTGTCGAAGCAATCAAGAAGTGGTACTTGGGTGGTTCTAGTGCTGTCTCCTCTTCCACCATCCAATCTTTGGCCTCTACTTTCTCTCAAGAATTATCCAAATGA |
| *SmCHIL*  (XM_024667149.1) | ATGGAAATGGACCCAACTTTTGCTCAATCCATCCAATCTCCATCTTCGTCTGAGACCTTGATTTTGTTGGGTCACGGTATCACAGACATGACCATTGAAACTATTCACGTCATCTTCACCAAGATTGGTGTTTACTTCGCCCCACAAGTTAAGGATCATTTGCAATCCTTCAAGTGTTTGCCAGTCTCCGAATTGTTGAAGGACGGTTCTGCTTTCTTCCAACAACTAATTCAAGCTCCAGTTTCTAAATTGATCAAGATCCTTTTAGTGAAGGGTCAATTGGGTTCTCAATACGCTTCCACTATCGAAACCTCTGTCAGAGACAGATTAGCTTACGATGACAAGTACGAAGAAGATGAAGAAATCGCTTTGGCCAACTTGTGTGAATTCTTTCAAAGTAAGAAGTTGGAACCAAACTCTACCATTGTCTATTCCTGGCCATCTTCCTCATCCCACGTCGAAGTTTTCGTCCACGAAGAAGGTTCCAAGGCTCCTTCTTCTTTCATCGTTAACAACGAAAACGTTAGCACCTCTATCATCGAATGGATTTTAGGTGAAAATTCCATGACTCCATCCACTGTTGAATCTGTTGCTAAATCTATTGCCACTGAATGTTGA |
| *OsCHIL*  (XM_015764458.3) | ATGGGTACTGAAATTGCTACCGTTGAAGTTGAAGGCATCCCATTCCCACAAGAAATTACTGTCTCCAAGCCATTGTCCTTGTTAGCTAACGGTATCACCGATATTGAAATCCATTTCTTGCAAATCAAATACAACGCCATTGGTGTCTATTTAGAAAAGGACAACGTTTTGGCTCACTTGGAATCTTGGAAGGGTAAGAAGGCTGAAGAATTGGTCCAAGATGATGGTTTCTTTCAAGCCTTGGTTTCTGCTCCAGTTGAAAAGTTGTTGAGAATTGTCGTCATCAAGGAAATCAAGGGTTCTCAATACGGTGTTCAATTGGAATCTTCTGTTCGTGACAGATTAGTTTCAGTTGACAAGTACGAAGAAGACGAAGAAGAAGCTCTAGAAAAGGTCACTGAATTTTTCCAATCCAAATACTTCAAGCCTAATTCCGTTATCACTTTCCACTTCCCAACCACTCCAGGTATTGCCGAGATCTCTTTCGTTACAGAAGGTAAGGGTGAAGCCAAATTGACCGTCGAAAACAAGAACGTTGCTGAAATGATTCAAAAGTGGTACTTGGGTGGTGAATCCGCTGTCTCTCCAACTACCGTCAAGAGTCTGGCTGACCAATTCGCTGCTTTGTTGTCTGCCTGA |

**Supplementary Table S5 Strains used in this study.**

| **Strain ID** | **Relevant genotype** | **Parental strain** | **Origin** |
| --- | --- | --- | --- |
| ***E. coli* strains** | | | |
| DH5α | *F^-^ ξ80lacZΔM15 Δ (ΔlacZYA-argF) U169 recA1 endA1 hsdR17 (rk^-^, mk^+^) phoA supE44 thi-1 gyrA96 relA1 λ^-^* |  | Lab store |
| BL21(DE3) | *F^-^ ompT gal dcm lon hsdSB (rB^-^, mB^-^) λ(DE3)* |  | Lab store |
| ***S. cerevisiae* strains** | | | |
| IMX581 | *MATa ura3-52 can1Δ::cas9-natNT2 TRP1 LEU2 HIS3* |  | Lab store |
| I15 | *MATa ura3-52 can1Δ::cas9-natNT2 TRP1 LEU2 HIS3 gal1/10/7Δ hmx1Δ XII-2::(CYC1t-At4CL1-Linker1-AtC4H-GAL2p)+(GAL1p-AtPAL2-TPS1t)+(FBA1t-CYB5-GAL10p)+(GAL1p-AtATR2-ADH1t) X-2::(GAL1p-GmCHS8-TPS1t)+(FBA1t-GmCHR5-GAL2p)+(GAL7p-GmCHI1B2-ADH1t) XI-2::(GAL2p-CrCPR2-ADH2t)+(TDH2t-Ge2HIS-GAL7p)+(GAL1p-GmHID-IDP1t)* |  | Lab store |
| ES01 | IMX581 with **p426GPD** | IMX581 | This study |
| ES02 | IMX581 with **pCG01** | IMX581 | This study |
| ES03 | IMX581 with **pCG02** | IMX581 | This study |
| ES04 | IMX581 with **pCG03** | IMX581 | This study |
| ES05 | IMX581 with **pCG04** | IMX581 | This study |
| ES06 | IMX581 with **pCG05** | IMX581 | This study |
| ES07 | IMX581 with **pCG06** | IMX581 | This study |
| ES08 | IMX581 with **pCG07** | IMX581 | This study |
| ES09 | IMX581 with **pCG08** | IMX581 | This study |
| ES10 | IMX581 with **pCG09** | IMX581 | This study |
| ES11 | IMX581 with **pCG10** | IMX581 | This study |
| ES12 | IMX581 with **pCG11** | IMX581 | This study |
| ES13 | IMX581 with **pCG12** | IMX581 | This study |
| ES14 | IMX581 with **pCG13** | IMX58 1 | This study |
| ES15 | IMX581 with **pCG14** | IMX581 | This study |
| ES16 | I15 with **pCG15** | I15 | This study |
| ES17 | I15 with **pCG16** | I15 | This study |
| ES18 | I15 with **pCG17** | I15 | This study |
| ES19 | I15 with **pCG18** | I15 | This study |
| ES20 | I15 with **pCG19** | I15 | This study |
| ES21 | I15 with **pCG20** | I15 | This study |
| P01 | *MATa ura3-52 can1Δ::cas9-natNT2 TRP1 LEU2 HIS3 gal1/10/7Δ hmx1Δ XII-2::(CYC1t-At4CL1-Linker1-AtC4H-GAL2p)+(GAL1p-AtPAL2-TPS1t)+(FBA1t-CYB5-GAL10p)+(GAL1p-AtATR2-ADH1t) X-2::(GAL1p-GmCHS8-TPS1t)+(FBA1t-GmCHR5-GAL2p)+(GAL7p-GmCHI1B2-ADH1t) XI-2::(GAL2p-CrCPR2-ADH2t)+(TDH2t-Ge2HIS-GAL7p)+(GAL1p-GmHID-IDP1t) elp3::(GAL1p-****Ge2HIS****-FBA1t)+(TPS1t-****GmHID****-GAL10p)* | I15 | This study |
| P02 | *MATa ura3-52 can1Δ::cas9-natNT2 TRP1 LEU2 HIS3 gal1/10/7Δ hmx1Δ XII-2::(CYC1t-At4CL1-Linker1-AtC4H-GAL2p)+(GAL1p-AtPAL2-TPS1t)+(FBA1t-CYB5-GAL10p)+(GAL1p-AtATR2-ADH1t) X-2::(GAL1p-GmCHS8-TPS1t)+(FBA1t-GmCHR5-GAL2p)+(GAL7p-GmCHI1B2-ADH1t) XI-2::(GAL2p-CrCPR2-ADH2t)+(TDH2t-Ge2HIS-GAL7p)+(GAL1p-GmHID-IDP1t) elp3::(GAL1p-Ge2HIS-FBA1t)+(TPS1t-GmHID-GAL10p) fas1::(GAL7p-****Ge2HIS****-pYX212t)+(IDP1t-****GmHID****-GAL2p)+(BGL2p-****FAS1****)* | P01 | This study |
| CN01 | *MATa ura3-52 can1Δ::cas9-natNT2 TRP1 LEU2 HIS3 gal1/10/7Δ hmx1Δ XII-2::(CYC1t-At4CL1-Linker1-AtC4H-GAL2p)+(GAL1p-AtPAL2-TPS1t)+(FBA1t-CYB5-GAL10p)+(GAL1p-AtATR2-ADH1t) X-2::(GAL1p-GmCHS8-TPS1t)+(FBA1t-GmCHR5-GAL2p)+(GAL7p-GmCHI1B2-ADH1t) XI-2::(GAL2p-CrCPR2-ADH2t)+(TDH2t-Ge2HIS-GAL7p)+(GAL1p-GmHID-IDP1t) elp3::(GAL1p-Ge2HIS- FBA1t)+(TPS1t-GmHID-GAL10p) fas1::(GAL7p-Ge2HIS-pYX212t)+(IDP1t-GmHID-GAL2p)+(BGL2p-FAS1) XII-1::(PRM5t-****PlOMT9****-SkGAL2p)+(SuGAL2p-****MtI3'H****-SPO1t)+(GAT2t-****AmUGT2****-SeGAL2p)* | P02 | This study |
| CN02 | *MATa ura3-52 can1Δ::cas9-natNT2 TRP1 LEU2 HIS3 gal1/10/7Δ hmx1Δ XII-2::(CYC1t-At4CL1-Linker1-AtC4H-GAL2p)+(GAL1p-AtPAL2-TPS1t)+(FBA1t-CYB5-GAL10p)+(GAL1p-AtATR2-ADH1t) X-2::(GAL1p-GmCHS8-TPS1t)+(FBA1t-GmCHR5-GAL2p)+(GAL7p-GmCHI1B2-ADH1t) XI-2::(GAL2p-CrCPR2-ADH2t)+(TDH2t-Ge2HIS-GAL7p)+(GAL1p-GmHID-IDP1t) elp3::(GAL1p-Ge2HIS-FBA1t)+(TPS1t-GmHID-GAL10p) fas1::(GAL7p-Ge2HIS-pYX212t)+(IDP1t-GmHID-GAL2p)+(BGL2p-FAS1) XII-1::(PRM5t-PlOMT9-SkGAL2p)+(SuGAL2p-MtI3'H-SPO1t)+(GAT2t-AmUGT2-SeGAL2p) XI-1::(CYC1t-****PlOMT9****-GAL1p)+(GAL7p-****MtI3'H****-HIS5t)+(FBA1t-****AmUGT2****-GAL10p)* | CN01 | This study |
| CN03 | *MATa ura3-52 can1Δ::cas9-natNT2 TRP1 LEU2 HIS3 gal1/10/7Δ hmx1Δ XII-2::(CYC1t-At4CL1-Linker1-AtC4H-GAL2p)+(GAL1p-AtPAL2-TPS1t)+(FBA1t-CYB5-GAL10p)+(GAL1p-AtATR2-ADH1t) X-2::(GAL1p-GmCHS8-TPS1t)+(FBA1t-GmCHR5-GAL2p)+(GAL7p-GmCHI1B2-ADH1t) XI-2::(GAL2p-CrCPR2-ADH2t)+(TDH2t-Ge2HIS-GAL7p)+(GAL1p-GmHID-IDP1t) elp3::(GAL1p-Ge2HIS-FBA1t)+(TPS1t-GmHID-GAL10p) fas1::(GAL7p-Ge2HIS-pYX212t)+(IDP1t-GmHID-GAL2p)+(BGL2p-FAS1) XII-1::(PRM5t-PlOMT9-SkGAL2p)+(SuGAL2p-MtI3'H-SPO1t)+(GAT2t-AmUGT2-SeGAL2p) XI-1::(CYC1t-PlOMT9-GAL1p)+(GAL7p-MtI3'H-HIS5t)+(FBA1t-AmUGT2-GAL10p) XI-3::(HSP26t-****PlOMT9****-SkGAL10p)+(SkGAL1p-****MtI3'H****-IDP1t)+(TIP1t-****AmUGT2****-SmGAL2p)* | CN02 | This study |
| PP01 | PL02 with **pCG20** | P02 | This study |
| SM01 | *MATa ura3-52 can1Δ::cas9-natNT2 TRP1 LEU2 HIS3 gal1/10/7Δ hmx1Δ XII-2::(CYC1t-At4CL1-Linker1-AtC4H-GAL2p)+(GAL1p-AtPAL2-TPS1t)+(FBA1t-CYB5-GAL10p)+(GAL1p-AtATR2-ADH1t) X-2::(GAL1p-GmCHS8-TPS1t)+(FBA1t-GmCHR5-GAL2p)+(GAL7p-GmCHI1B2-ADH1t) XI-2::(GAL2p-CrCPR2-ADH2t)+(TDH2t-Ge2HIS-GAL7p)+(GAL1p-GmHID-IDP1t) elp3::(GAL1p-Ge2HIS-FBA1t)+(TPS1t-GmHID-GAL10p) fas1::(GAL7p-Ge2HIS-pYX212t)+(IDP1t-GmHID-GAL2p)+(BGL2p-FAS1) XII-3::(CYC1t-****MET6****-tHXT7p)* | P02 | This study |
| SM02 | *MATa ura3-52 can1Δ::cas9-natNT2 TRP1 LEU2 HIS3 gal1/10/7Δ hmx1Δ XII-2::(CYC1t-At4CL1-Linker1-AtC4H-GAL2p)+(GAL1p-AtPAL2-TPS1t)+(FBA1t-CYB5-GAL10p)+(GAL1p-AtATR2-ADH1t) X-2::(GAL1p-GmCHS8-TPS1t)+(FBA1t-GmCHR5-GAL2p)+(GAL7p-GmCHI1B2-ADH1t) XI-2::(GAL2p-CrCPR2-ADH2t)+(TDH2t-Ge2HIS-GAL7p)+(GAL1p-GmHID-IDP1t) elp3::(GAL1p-Ge2HIS-FBA1t)+(TPS1t-GmHID-GAL10p) fas1::(GAL7p-Ge2HIS-pYX212t)+(IDP1t-GmHID-GAL2p)+(BGL2p-FAS1) XII-3::(CYC1t-****SAM2****-tHXT7p)* | P02 | This study |
| SM03 | *MATa ura3-52 can1Δ::cas9-natNT2 TRP1 LEU2 HIS3 gal1/10/7Δ hmx1Δ XII-2::(CYC1t-At4CL1-Linker1-AtC4H-GAL2p)+(GAL1p-AtPAL2-TPS1t)+(FBA1t-CYB5-GAL10p)+(GAL1p-AtATR2-ADH1t) X-2::(GAL1p-GmCHS8-TPS1t)+(FBA1t-GmCHR5-GAL2p)+(GAL7p-GmCHI1B2-ADH1t) XI-2::(GAL2p-CrCPR2-ADH2t)+(TDH2t-Ge2HIS-GAL7p)+(GAL1p-GmHID-IDP1t) elp3::(GAL1p-Ge2HIS-FBA1t)+(TPS1t-GmHID-GAL10p) fas1::(GAL7p-Ge2HIS-pYX212t)+(IDP1t-GmHID-GAL2p)+(BGL2p-FAS1) XII-3::(CYC1t-****MET6****-tHXT7p)+(TDH3p-****SAM2****-HIS5t)* | P02 | This study |
| SM04 | *MATa ura3-52 can1Δ::cas9-natNT2 TRP1 LEU2 HIS3 gal1/10/7Δ hmx1Δ XII-2::(CYC1t-At4CL1-Linker1-AtC4H-GAL2p)+(GAL1p-AtPAL2-TPS1t)+(FBA1t-CYB5-GAL10p)+(GAL1p-AtATR2-ADH1t) X-2::(GAL1p-GmCHS8-TPS1t)+(FBA1t-GmCHR5-GAL2p)+(GAL7p-GmCHI1B2-ADH1t) XI-2::(GAL2p-CrCPR2-ADH2t)+(TDH2t-Ge2HIS-GAL7p)+(GAL1p-GmHID-IDP1t) elp3::(GAL1p-Ge2HIS-FBA1t)+(TPS1t-GmHID-GAL10p) fas1::(GAL7p-Ge2HIS-pYX212t)+(IDP1t-GmHID-GAL2p)+(BGL2p-FAS1) XII-4::(CYC1t-****MET6****-tHXT7p)+(TDH3p-****SAM2****-HIS5t)+(FBA1t-****MET13^S443F^****-PGK1p)* | P02 | This study |
| PS01 | SM01 with **pCG20** | SM01 | This study |
| PS02 | SM02 with **pCG20** | SM02 | This study |
| PS03 | SM03 with **pCG20** | SM03 | This study |
| PS04 | SM04 with **pCG20** | SM04 | This study |
| SM05 | *MATa ura3-52 can1Δ::cas9-natNT2 TRP1 LEU2 HIS3 gal1/10/7Δ hmx1Δ XII-2::(CYC1t-At4CL1-Linker1-AtC4H-GAL2p)+(GAL1p-AtPAL2-TPS1t)+(FBA1t-CYB5-GAL10p)+(GAL1p-AtATR2-ADH1t) X-2::(GAL1p-GmCHS8-TPS1t)+(FBA1t-GmCHR5-GAL2p)+(GAL7p-GmCHI1B2-ADH1t) XI-2::(GAL2p-CrCPR2-ADH2t)+(TDH2t-Ge2HIS-GAL7p)+(GAL1p-GmHID-IDP1t) elp3::(GAL1p-Ge2HIS-FBA1t)+(TPS1t-GmHID-GAL10p) fas1::(GAL7p-Ge2HIS-pYX212t)+(IDP1t-GmHID-GAL2p)+(BGL2p-FAS1) XII-3::(CYC1t-****PGM2****-tHXT7p)+(TDH3p-****UGP1****-HIS5t)* | P02 | This study |
| SM06 | *MATa ura3-52 can1Δ::cas9-natNT2 TRP1 LEU2 HIS3 gal1/10/7Δ hmx1Δ XII-2::(CYC1t-At4CL1-Linker1-AtC4H-GAL2p)+(GAL1p-AtPAL2-TPS1t)+(FBA1t-CYB5-GAL10p)+(GAL1p-AtATR2-ADH1t) X-2::(GAL1p-GmCHS8-TPS1t)+(FBA1t-GmCHR5-GAL2p)+(GAL7p-GmCHI1B2-ADH1t) XI-2::(GAL2p-CrCPR2-ADH2t)+(TDH2t-Ge2HIS-GAL7p)+(GAL1p-GmHID-IDP1t) elp3::(GAL1p-Ge2HIS-FBA1t)+(TPS1t-GmHID-GAL10p) fas1::(GAL7p-Ge2HIS-pYX212t)+(IDP1t-GmHID-GAL2p)+(BGL2p-FAS1) XII-3::(CYC1t-****PGM2****-tHXT7p)+(TDH3p-****UGP1****-HIS5t)+(FBA1t-****URA6****-TEF1p)+(PGK1p-****YNK1****-IDP1t)* | P02 | This study |
| SM07 | *MATa ura3-52 can1Δ::cas9-natNT2 TRP1 LEU2 HIS3 gal1/10/7Δ hmx1Δ XII-2::(CYC1t-At4CL1-Linker1-AtC4H-GAL2p)+(GAL1p-AtPAL2-TPS1t)+(FBA1t-CYB5-GAL10p)+(GAL1p-AtATR2-ADH1t) X-2::(GAL1p-GmCHS8-TPS1t)+(FBA1t-GmCHR5-GAL2p)+(GAL7p-GmCHI1B2-ADH1t) XI-2::(GAL2p-CrCPR2-ADH2t)+(TDH2t-Ge2HIS-GAL7p)+(GAL1p-GmHID-IDP1t) elp3::(GAL1p-Ge2HIS-FBA1t)+(TPS1t-GmHID-GAL10p) fas1::(GAL7p-Ge2HIS-pYX212t)+(IDP1t-GmHID-GAL2p)+(BGL2p-FAS1) XII-4::(CYC1t-MET6-tHXT7p)+(TDH3p-SAM2-HIS5t)+(FBA1t-MET13^S443F^-PGK1p) XII-3::(CYC1t-****PGM2-****tHXT7p)+(TDH3p-****UGP1****-HIS5t)+(FBA1t-****URA6****-TEF1p)+(PGK1p-****YNK1****-IDP1t)* | SM04 | This study |
| PS05 | SM05 with **pCG20** | SM05 | This study |
| PS06 | SM06 with **pCG20** | SM06 | This study |
| PS07 | SM07 with **pCG20** | SM07 | This study |
| SM08 | *MATa ura3-52 can1Δ::cas9-natNT2 TRP1 LEU2 HIS3 gal1/10/7Δ hmx1Δ XII-2::(CYC1t-At4CL1-Linker1-AtC4H-GAL2p)+(GAL1p-AtPAL2-TPS1t)+(FBA1t-CYB5-GAL10p)+(GAL1p-AtATR2-ADH1t) X-2::(GAL1p-GmCHS8-TPS1t)+(FBA1t-GmCHR5-GAL2p)+(GAL7p-GmCHI1B2-ADH1t) XI-2::(GAL2p-CrCPR2-ADH2t)+(TDH2t-Ge2HIS-GAL7p)+(GAL1p-GmHID-IDP1t) elp3::(GAL1p-Ge2HIS-FBA1t)+(TPS1t-GmHID-GAL10p) fas1::(GAL7p-Ge2HIS-pYX212t)+(IDP1t-GmHID-GAL2p)+(BGL2p-FAS1) XII-3::(TDH3p-****GmCHIL****-CYC1t)* | P02 | This study |
| SM09 | *MATa ura3-52 can1Δ::cas9-natNT2 TRP1 LEU2 HIS3 gal1/10/7Δ hmx1Δ XII-2::(CYC1t-At4CL1-Linker1-AtC4H-GAL2p)+(GAL1p-AtPAL2-TPS1t)+(FBA1t-CYB5-GAL10p)+(GAL1p-AtATR2-ADH1t) X-2::(GAL1p-GmCHS8-TPS1t)+(FBA1t-GmCHR5-GAL2p)+(GAL7p-GmCHI1B2-ADH1t) XI-2::(GAL2p-CrCPR2-ADH2t)+(TDH2t-Ge2HIS-GAL7p)+(GAL1p-GmHID-IDP1t) elp3::(GAL1p-Ge2HIS-FBA1t)+(TPS1t-GmHID-GAL10p) fas1::(GAL7p-Ge2HIS-pYX212t)+(IDP1t-GmHID-GAL2p)+(BGL2p-FAS1) XII-3::(TDH3p-****SmCHIL****-CYC1t)* | P02 | This study |
| SM10 | *MATa ura3-52 can1Δ::cas9-natNT2 TRP1 LEU2 HIS3 gal1/10/7Δ hmx1Δ XII-2::(CYC1t-At4CL1-Linker1-AtC4H-GAL2p)+(GAL1p-AtPAL2-TPS1t)+(FBA1t-CYB5-GAL10p)+(GAL1p-AtATR2-ADH1t) X-2::(GAL1p-GmCHS8-TPS1t)+(FBA1t-GmCHR5-GAL2p)+(GAL7p-GmCHI1B2-ADH1t) XI-2::(GAL2p-CrCPR2-ADH2t)+(TDH2t-Ge2HIS-GAL7p)+(GAL1p-GmHID-IDP1t) elp3::(GAL1p-Ge2HIS-FBA1t)+(TPS1t-GmHID-GAL10p) fas1::(GAL7p-Ge2HIS-pYX212t)+(IDP1t-GmHID-GAL2p)+(BGL2p-FAS1) XII-3::(TDH3p-****OsCHIL****-CYC1t)* | P02 | This study |
| PS08 | SM08 with **pCG20** | SM08 | This study |
| PS09 | SM09 with **pCG20** | SM09 | This study |
| PS10 | SM10 with **pCG20** | SM10 | This study |
| PM01 | PL02 with **pCG21** | P02 | This study |
| PM02 | PL02 with **pCG22** | P02 | This study |

**Supplementary Table S6 Primers used in this study.**

| **Primer ID** | **Name** | **Sequence (5′-3′)** |
| --- | --- | --- |
| **Primers for plasmid-expression and chromosomal integration DNA cassettes** | | |
| P1 | p426GPD backbone-F1 | CGGTACCCAATTCGCCCTATAG |
| P2 | p426GPD backbone-F2 (with *CYC1t*)^a^ | AAGCTTATCGATACCGTCG |
| P3 | p426GPD backbone-R1 | AGCTCCAGCTTTTGTTCCCT |
| P4 | p426GPD backbone-R2 (with *TDH3p*) | GGATCCACTAGTTCTAGAATCC |
| P5 | Seq-p426GPD-F | CAATTAATGTGAGTTACCTCACTCA |
| P6 | Seq-p426GPD-R | GCGATCGGTGCGGG |
| P7 | *ELP3 us*-F | TTGTCCAGTTTCTCAAGCAG |
| P8 | *ELP3 us*-R (with *GAL1p*-F) | GAGGGCTGTCACCCGCTCGGCGGCTTCTAATCCGCTTTGTCAGGGTGTTCTTCG |
| P9 | *ELP3 ds*-F (with *GAL10p*-F) | GTAATACGCTTAACTGCTCATTGCTATATTGAAGGTAAATAGAACTTTTATGTGC |
| P10 | *ELP3 ds*-R | CTTCGTCGTGTATTTGCAATAT |
| P11 | *FAS1p*-F | GCTATTTAACGCTTTTGGTCTG |
| P12 | *FAS1p*-R (with *GAL7p*-F) | GCATATTTTCAAGAAGGATAGTAAGCTGGCAAAATATTTTTCAGAAATATTCAAAGAGC |
| P13 | *FAS1* CDS-F | TTCGCTCATTATGGACGCTTA |
| P14 | *FAS1* CDS-R | CAAAGTAGTCGTCGGTGTTAC |
| P15 | *XII-1 us*-F | GTTGAGCTCTGTCCTTCATGG |
| P16 | *XII-1 us*-R (with *PRM5t*-R) | TTATTATATTATTTGTTTAGTCTCTCTTTTTGGGTTCTATGAAAGAACCGAACCGATGC |
| P17 | *XII-1 ds*-F (with *SeGAL2p*-F) | GTGATCTGGATCTTGTAATCTCCTGTTCTCTGTGGGTTCAGTTTAGTGCTCTGTCTGAG |
| P18 | *XII-1 ds*-R | TGATGACTGTTTCTCAATCTTTATG |
| P19 | *XI-1 us*-F | ATTTGTGTGAAGGAATAGTGACG |
| P20 | *XI-1 us*-R (with *CYC1t*-R) | GGACGCTCGAAGGCTTTAATTTGCGGCCGGTACCCAATGGGCTTGGTATTCCGTG |
| P21 | *XI-1 ds*-F (with *GAL10p*-F) | TCAGTAATACGCTTAACTGCTCATTGCTATATTGATTTCTTGGCATTGGCAAATC |
| P22 | *XI-1 ds*-R | AAGAGCCGAGTCCCCATCAG |
| P23 | *XI-3 us*-F | AGTTACTTGCTCTATGCGTTTGC |
| P24 | *XI-3 us*-R (with *HSP26t*-R) | TTATTTTTGACTAAGTTTTAACGAAGGAATCTAACCTCGTAATCAGACGCACGCTTGGC |
| P25 | *XI-3 ds*-F (with *SmGAL2p*-F) | CATCGTGATTCAGATCTGGAGAACTCCTCTGATCCGTAATTACGTGGATTGAGCCAGCA |
| P26 | *XI-3 ds*-R | TGAGAATCCGGACCAGCAGAT |
| P27 | *XII-3 us*-F | TGTGCCCCTTAAAATTCATATAC |
| P28 | *XII-3 us*-R1 (with *CYC1t*-R) | TTTGGGACGCTCGAAGGCTTTAATTTGCGGCCGGTACCCGAATGAGCAGGTACCCCTTA |
| P29 | *XII-3 us*-R2 (with *TDH3p*-F) | TGAAATGGCGAGTATTGATAATGATAAACTGGAATGAGCAGGTACCCCTTATTATAATG |
| P30 | *XII-3 ds*-F1 (with *tHXT7p*-F) | GAAGAACACGCAGGGGCCCGAAATTGTTCCTACGAGGCATAGAGCTAATTAGGTTTGAG |
| P31 | *XII-3 ds*-F2 (with *HIS5t*-R) | GCGCCACTTCTATAAAAGGTCTCATGATATTGTTACGCATAGAGCTAATTAGGTTTGAG |
| P32 | *XII-3 ds*-F3 (with *IDP1t*-R) | CTTATCAAGGTTCCCCAAGTTCGGATCATTACCATCGCATAGAGCTAATTAGGTTTGAG |
| P33 | *XII-3 ds*-F4 (with *CYC1t*-R) | TTTGGGACGCTCGAAGGCTTTAATTTGCGGCGCATAGAGCTAATTAGGTTTGAGTTAAG |
| P34 | *XII-3 ds*-R | GAACTTACAAGCTGATTTTGGT |
| P35 | *XII-4 us*-F | GTATCCGGCTGTTCCTTCATAG |
| P36 | *XII-4 us*-R (with *CYC1t*-R) | GGGACGCTCGAAGGCTTTAATTTGCGGCCGGTACCCTGCCATAGTATGTGTGATGGAAA |
| P37 | *XII-4 ds*-F (with *PGK1p*-F) | AAGAACACGCAGGGGCCCGAAATTGTTCCTACGAGATTCCCCATTAGAGTCAAATAAAA |
| P38 | *XII-4 ds*-R | TTTCTGCCGTACCTGGATGGTC |
| P39 | *GAL1p*-F1 (with *GAL7p*-F) | GATATAGAGTGCATATTTTCAAGAAGGATAGTAAGCTGGCAAACGGATTAGAAGCCGCC |
| P40 | *GAL1p*-F2 | CGGATTAGAAGCCGCC |
| P41 | *GAL1p*-R | TTTGTTCTCCTTGACGTTAAAG |
| P42 | *GAL7p*-F | TTTGCCAGCTTACTATCCTTCTTG |
| P43 | *GAL7p*-R | TTTGTTTTTTGAGGGAATATTC |
| P44 | *GAL10p*-F | TCAATATAGCAATGAGCAGTTAAG |
| P45 | *GAL10p*-R | TTTGTTTTTCAAAAATTCTTACTTTTT |
| P46 | *GAL2p*-F | CAGAAGGCACATCTATTACATTTAC |
| P47 | *GAL2p*-R | TTTGTTTATGAAAGAATTATTTTTTTTATTATG |
| P48 | *SkGAL2p*-F (with *SuGAL2p*-F) | GCCAACAAATTAAGGTTTTTGGAAAGCCCCTTCTTTTATTTAAGGAGGTTTTACGGACC |
| P49 | *SkGAL2p*-R | TGTTTTTATGAAAAAATTATTTTATATTATGTTAATCT |
| P50 | *SuGAL2p*-F | AAAAGAAGGGGCTTTCCAAA |
| P51 | *SuGAL2p*-R | TGTTTTTTTTTATCTATTCTTCAAAAAAATCTT |
| P52 | *SeGAL2p*-F | CCACAGAGAACAGGAGATTACA |
| P53 | *SeGAL2p*-R | TGTTTTTGTAAATGTGTGTATATATTATATTATAGTAT |
| P54 | *SkGAL10p*-R | ATGTTTTATTTGTAGAGTTCAATTTTTC |
| P55 | *SkGAL1p*-R | TGTTTTTATAGATTTTTATTTTTATCTTTTATGAGT |
| P56 | *SmGAL2p*-F | TTACGGATCAGAGGAGTTCT |
| P57 | *SmGAL2p*-R | TGTTTTTATGAAAAAGTTTTTTATATGTTATTCT |
| P58 | *tHXT7p*-F1 | CTCGTAGGAACAATTTCGGG |
| P59 | *tHXT7p*-F2 (with *TDH3p*-F) | TATTCTTTGAAATGGCAGTATTGATAATGATAAACTCGACTCGTAGGAACAATTTCGGG |
| P60 | *tHXT7p*-R | TTTTTGATTAAAATTAAAAAAACTTTTTG |
| P61 | *TDH3p*-F | TCGAGTTTATCATTATCAATACTGCC |
| P62 | *TDH3p*-R | TTTGTTTGTTTATGTGTGTTTATTCGA |
| P63 | *PGK1p*-F | ACGCACAGATATTATAACATCTGC |
| P64 | *PGK1p*-R | TTTGTTATATTTGTTGTAAAAAGTAGATAA |
| P65 | *BGL2p*-F (with *GAL2p*-F) | GCTCAGTAAATGTAATAGATGTGCCTTCTGAATAAATAGCAATTAATAACTGATATTAC |
| P66 | *BGL2p*-R (with *FAS1* CDS-F) | GTCTTGTGGAGTAAGCGTCCATAATGAGCGAACTTTGACCGTTTTTCTTTTTG |
| P67 | *CYC1t*-F | GATACCGTCGACCTCGAGTC |
| P68 | *CYC1t*-R | GGGTACCGGCCGCAAATTAA |
| P69 | *HIS5t*-F | ATAGATTAATTTAAACAGTATATGTAC |
| P70 | *HIS5t*-R1 (with *FBA1t*-R) | CTTTGTAAAGTCTTTCATAGTAGCTTACTGTAACAATATCATGAGACCTTTTATAGAAG |
| P71 | *HIS5t*-R2 | GTAACAATATCATGAGACCTTTTATAG |
| P72 | *FBA1t*-F | GTTAATTCAAATTAATTGATATAGTTT |
| P73 | *FBA1t*-R | AGTAAGCTACTATGAAAGACTTTAC |
| P74 | *TPS1t*-F | TGAACCCGATGCAAATGAGAC |
| P75 | *TPS1t*-R (with *FBA1t*-R) | CTTCGAGTTCTTTGTAAAGTCTTTCATAGTAGCTTACTTGTTTCGAAGAAGAGATCAGC |
| P76 | *pYX212t*-F | TAGGGCCCACAAGCTTAC |
| P77 | *pYX212t*-R (with *IDP1t*-R) | TTCCTTATCAAGGTTCCCCAAGTTCGGATCATTACCATCGCCGTAAACCACTAAATCGG |
| P78 | *IDP1t*-F | TCGAATTTACGTAGCCCAATC |
| P79 | *IDP1t*-R1 | GATGGTAATGATCCGAACTTGG |
| P80 | *IDP1t*-R2 (with *TIP1t*-R) | ACGTTCTCTTTTTCGAGATTAGTGCTTCTTCCCAATCGATGGTAATGATCCGAACTTGG |
| P81 | *PRM5t*-F | AAACTTTTATGATATTTTGCAATA |
| P82 | *PRM5t*-R | ATAGAACCCAAAAAGAGAGAC |
| P83 | *SPO1t*-F | CAAAGACGTTGTTTCATCGC |
| P84 | *SPO1t*-R (with *GAT2t*-R) | TATTTAGCCGCCCGCGTTTTCCTAACGTTTTCCCGCTTATTTTCTGCCGAATTTTCATG |
| P85 | *GAT2t*-F | TATAAAATCATACATTCATATAATATC |
| P86 | *GAT2t*-R | GGGAAAACGTTAGGAAAACGC |
| P87 | *HSP26t*-F | AGTGACCTGGCTCTATAGTG |
| P88 | *HSP26t*-R | ACGAGGTTAGATTCCTTCGTT |
| P89 | *TIP1t*-F | AAGGGAACCTTTTACAACAAATA |
| P90 | *TIP1t*-R | GATTGGGAAGAAGCACTAATC |
| P91 | *PlOMT9*-F1 (with *TDH3p*-R) | CGACGGATTCTAGAACTAGTATGGGTTCTAACAACGGTAG |
| P92 | *PlOMT9*-F2 (with *GAL1p*-R) | ATATACCTCTATACTTTAACGTCAAGGAGAACAAAATGGGTTCTAACAACGGTAG |
| P93 | *PlOMT9*-F3 (with *SkGAL2p*-R) | AAGATTAACATAATATAAAATAATTTTTTCATAAAAACAATGGGTTCTAACAACGGTAG |
| P94 | *PlOMT9*-F4 (with *SkGAL10p*-R) | GAAGTAAGAGAGAAAAATTGAACTCTACAAATAAAACATATGGGTTCTAACAACGGTAG |
| P95 | *PlOMT9*-R1 (with *CYC1t*-F) | TCGAGGTCGACGGTATCGATTTAGGGGTAGATCTCAATCAAAG |
| P96 | *PlOMT9*-R2 (with *PRM5t*-F) | ACTGCTTAAAAAAAATATTGCAAAATATCATAAAAGTTTTTAGGGGTAGATCTCAATCA |
| P97 | *PlOMT9*-R3 (with *HSP26t*-F) | GTCCTCGCGAGAGGGACAACACTATAGAGCCAGGTCACTTTAGGGGTAGATCTCAATCA |
| P98 | *AmOMT*-F (with *TDH3p*-R) | CGACGGATTCTAGAACTAGTATGGGTTCTAGGTACGTCCAAAAAG |
| P99 | *AmOMT*-R (with *CYC1t*-F) | TCGAGGTCGACGGTATCGATTCATGGGTAGACCTCAATCAAAG |
| P100 | *MsOMT8*-F (with *TDH3p*-R) | CGACGGATTCTAGAACTAGTATGGCCTCTTCTATTAACGGTAG |
| P101 | *MsOMT8*-R (with *CYC1t*-F) | TCGAGGTCGACGGTATCGATTCATGGGTAGATCTCGATCAAAG |
| P102 | *GmOMT2*-F (with *TDH3p*-R) | CGACGGATTCTAGAACTAGTATGGCCTCTTCTTTGAACAAC |
| P103 | *GmOMT2*-R (with *CYC1t*-F) | TCGAGGTCGACGGTATCGATTCAGGGGTAGATCTCAATCAAAG |
| P104 | *AmI3'H*-F1 (with *TDH3p*-R) | CGACGGATTCTAGAACTAGTATGGCTCCCTTGTTGTATTAC |
| P105 | *AmI3'H*-F2 (with *GAL7p*-R) | AAAAAAACAGTTGAATATTCCCTCAAAAAACAAAATGGCTCCCTTGTTGTATTAC |
| P106 | *AmI3'H*-R1 (with *CYC1t*-F) | TCGAGGTCGACGGTATCGATTCATTTCATAATGTCATTGACAATTG |
| P107 | *AmI3'H*-R2 (with *HIS5t*-F) | CTGTACATATACTGTTTAAATTAATCTATTCATTTCATAATGTCATTGACAATTG |
| P108 | *MtI3'H*-F1 (with *TDH3p*-R) | AACTTAGTTTCGACGGATTCTAGAACTAGTATGGCTTTGTTCTACTACTCC |
| P109 | *MtI3'H*-F2 (with *GAL7p*-R) | AAAAAAAACAGTTGAATATTCCCTCAAAAAACAAAATGGCTTTGTTCTACTACTC |
| P110 | *MtI3'H*-F3 (with *SuGAL2p*-R) | AAAGAGAAGATTTTTTTGAAGAATAGATAAAAAAAAACAATGGCTTTGTTCTACTACTC |
| P111 | *MtI3'H*-F4 (with *SkGAL1p*-R) | ATTACTCATAAAAGATAAAAATAAAAATCTATAAAAACAATGGCTTTGTTCTACTACTC |
| P112 | *MtI3'H*-R1 (with *CYC1t*-F) | ATTACATGACTCGAGGTCGACGGTATCGATTCATTTGGTGACGTCGTTAG |
| P113 | *MtI3'H*-R2 (with *HIS5t*-F) | ATAAAACTGTACATATACTGTTTAAATTAATCTATTCATTTGGTGACGTCGTTAG |
| P114 | *MtI3'H*-R3 (with *SPO1t*-F) | TAACCTTCTTGGTAATAGCGCGATGAAACAACGTCTTTGTCATTTGGTGACGTCGTTAG |
| P115 | *MtI3'H*-R4 (with *IDP1t*-F) | TGAAAAAAAAAAGTGGTAGATTGGGCTACGTAAATTCGATCATTTGGTGACGTCGTTAG |
| P116 | *ThF3'H*-F (with *TDH3p*-R) | CGACGGATTCTAGAACTAGTATGTCTCCATTGGCTTTGATG |
| P117 | *ThF3'H*-R (with *CYC1t*-F) | TCGAGGTCGACGGTATCGATTCAGTAGACATGAGTAGCCAATC |
| P118 | *GmUGT*-F1 (with *TDH3p*-R) | CGACGGATTCTAGAACTAGTATGACCATGAAGGACTCTATCG |
| P119 | *GmUGT*-F2 (with *GAL10p*-R) | TCCAAAAAAAAAGTAAGAATTTTTGAAAAACAAAATGACCATGAAGGACTCTATC |
| P120 | *GmUGT*-R1 (with *CYC1t*-F) | TCGAGGTCGACGGTATCGATTCAGTGTTCTCTCCACAACTC |
| P121 | *GmUGT*-R2 (with *FBA1t*-F) | CATTAAAAAACTATATCAATTAATTTGAATTAACTCAGTGTTCTCTCCACAACTC |
| P122 | *GuUGT6*-F1 (with *TDH3p*-R) | CGACGGATTCTAGAACTAGTATGAAAGACACTATCATCTTGTATCC |
| P123 | *GuUGT6*-F2 (with *GAL10p*-R) | AAAAAAAAGTAAGAATTTTTGAAAAACAAAATGAAAGACACTATCATCTTGTATC |
| P124 | *GuUGT6*-R1 (with *CYC1t*-F) | TCGAGGTCGACGGTATCGATTCATTGCCAATGCAAGTTGAATG |
| P125 | *GuUGT6*-R2 (with *FBA1t*-F) | TTAAAAAACTATATCAATTAATTTGAATTAACTCATTGCCAATGCAAGTTGAATG |
| P126 | *ApUGT3*-F (with *TDH3p*-R) | CGACGGATTCTAGAACTAGTATGGGTTACCACTCTCATATTG |
| P127 | *ApUGT3*-R (with *CYC1t*-F) | TCGAGGTCGACGGTATCGATTCACTTAGAGATACCGATGATC |
| P128 | *AmUGT1*-F (with *TDH3p*-R) | CGACGGATTCTAGAACTAGTATGGAGTCTAAGACCGACTC |
| P129 | *AmUGT1*-R (with *CYC1t*-F) | TCGAGGTCGACGGTATCGATTCAAACCTGAGAAGTGAACC |
| P130 | *AmUGT2*-F1 (with *TDH3p*-R) | CGACGGATTCTAGAACTAGTATGAAAGACACTTTGGTTCTATATC |
| P131 | *AmUGT2*-F2 (with *GAL10p*-R) | ATCCAAAAAAAAAGTAAGAATTTTTGAAAAACAAAATGAAAGACACTTTGGTTCT |
| P132 | *AmUGT2*-F3 (with *SeGAL2p*-R) | AATACTATAATATAATATATACACACATTTACAAAAACAATGAAAGACACTTTGGTTCT |
| P133 | *AmUGT2*-F4 (with *SmGAL2p*-R) | AATAAAGAATAACATATAAAAAACTTTTTCATAAAAACAATGAAAGACACTTTGGTTCT |
| P134 | *AmUGT2*-R1 (with *CYC1t*-F) | TCGAGGTCGACGGTATCGATCTATCTAAACAAGAATGGAGAATTTGG |
| P135 | *AmUGT2*-R2 (with *FBA1t*-F) | TAAAAAACTATATCAATTAATTTGAATTAACCTATCTAAACAAGAATGGAGAATT |
| P136 | *AmUGT2*-R3 (with *GAT2t*-F) | ACGTAATGGATATTATATGAATGTATGATTTTATACTATCTAAACAAGAATGGAGAATT |
| P137 | *AmUGT2*-R4 (with *TIP1t*-F) | TAATTTTTCAAATATTTGTTGTAAAAGGTTCCCTTCTATCTAAACAAGAATGGAGAATT |
| P138 | *AmUGT3*-F (with *TDH3p*-R) | CGACGGATTCTAGAACTAGTATGAAGGACACTATTGTCTTGTATC |
| P139 | *AmUGT3*-R (with *CYC1t*-F) | TCGAGGTCGACGGTATCGATTTATTGTTGCCTCCAGGAGTC |
| P140 | *AmUGT4*-F (with *TDH3p*-R) | CGACGGATTCTAGAACTAGTATGGCTGTCAACGGTATGAAG |
| P141 | *AmUGT4*-R (with *CYC1t*-F) | TCGAGGTCGACGGTATCGATTCAGTTCCTACCAATAATATTGTC |
| P142 | *Ge2HIS*-F1 (with *GAL1p*-R) | ATACCTCTATACTTTAACGTCAAGGAGAACAAAATGTTGGTTGAATTGGCTATCAC |
| P143 | *Ge2HIS*-F2 (with *GAL7p*) | AAAAAAACAGTTGAATATTCCCTCAAAAAACAAAATGTTGGTTGAATTGGCTATCAC |
| P144 | *Ge2HIS*-R1 (with *FBA1t*-F) | ATTAAAAAACTATATCAATTAATTTGAATTAACTTATGAAGAAAACAATTTTGGAACTG |
| P145 | *Ge2HIS*-R2 (with *pYX212t*-F) | TACCCGGGTCGACGCGTAAGCTTGTGGGCCCTATTATGAAGAAAACAATTTTGGAACTG |
| P146 | *GmHID*-F1 (with *GAL10p*-R) | CCAAAAAAAAAGTAAGAATTTTTGAAAAACAAAATGGCTAAGGAAATCGTTAAG |
| P147 | *GmHID*-F2 (with *GAL2p*-R) | CATAATAAAAAAAATAATTCTTTCATAAACAAAATGGCTAAGGAAATCGTTAAG |
| P148 | *GmHID*-R1 (with *TPS1t*-F) | GAATAGACGATCGTCTCATTTGCATCGGGTTCATTAAACCAAAAATGAAGCTAATC |
| P149 | *GmHID*-R2 (with *IDP1t*-F) | AAAAAAAGTGGTAGATTGGGCTACGTAAATTCGATTAAACCAAAAATGAAGCTAATC |
| P150 | *MET6*-F (with *tHXT7p*-R) | CACAAAAACAAAAAGTTTTTTTAATTTTAATCAAAAAATGGTTCAATCTGCTGTCTTAG |
| P151 | *MET6*-R (with *CYC1t*-F) | ATAACTAATTACATGACTCGAGGTCGACGGTATCTTAATTCTTGTATTGTTCACGGAAG |
| P152 | *SAM2*-F (with *tHXT7p*-R) | CACAAAAACAAAAAGTTTTTTTAATTTTAATCAAAAAATGGTTCAATCTGCTGTCTTAG |
| P153 | *SAM2*-R (with *CYC1t*-F) | AACTAATTACATGACTCGAGGTCGACGGTATCTTAAAATTCCAATTTCTTTGGTTTTTC |
| P154 | *SAM2*-F (with *TDH3p*-R) | AGAACTTAGTTTCGAATAAACACACATAAACAAACAAAATGTCCAAGAGCAAAACTTTC |
| P155 | *SAM2*-R (with *HIS5t*-F) | AAACTGTACATATACTGTTTAAATTAATCTATTTAAAATTCCAATTTCTTTGGTTTTTC |
| P156 | *MET13^S443F^*-F (with *PGK1p*-R) | GTAATTATCTACTTTTTACAACAAATATAACAAAATGAAGATCACAGAAAAATTAGAGC |
| P157 | *MET13^S443F^*-R (with *FBA1t*-F) | TCATTAAAAAACTATATCAATTAATTTGAATTAACTTATAGGCTTAGTAGGATGGAATG |
| P158 | *PGM2*-F (with *tHXT7p*-R) | CACAAAAACAAAAAGTTTTTTTAATTTTAATCAAAAAATGTCATTTCAAATTGAAACGG |
| P159 | *PGM2*-R (with *CYC1t*-F) | TGACATAACTAATTACATGACTCGAGGTCGACGGTATCTTAAGTACGAACCGTTGGTTC |
| P160 | *UGP1*-F (with *TDH3p*-R) | AGAACTTAGTTTCGAATAAACACACATAAACAAACAAAATGTCCACTAAGAAGCACACC |
| P161 | *UGP1*-R (with *HIS5t*-F) | AAAACTGTACATATACTGTTTAAATTAATCTATTCAATGTTCCAAGATTTGCAAATTAC |
| P162 | *URA6*-F (with *TEF1p*-R) | AAGAAAGCATAGCAATCTAATCTAAGTTTTAATTACAAAATGACAGCTGCCACTACATC |
| P163 | *URA6*-R (with *FBA1t*-F) | TCATTAAAAAACTATATCAATTAATTTGAATTAACCTATAAGCTATCACGGATAGCGTC |
| P164 | *YNK1*-F (with *PGK1p*-R) | AAGTAATTATCTACTTTTTACAACAAATATAACAAAATGTCTAGTCAAACAGAAAGAAC |
| P165 | *YNK1*-R (with *IDP1t*-F) | AAAAAAAAGTGGTAGATTGGGCTACGTAAATTCGATCATTCATAAATCCACTTAGCTTG |
| P166 | *GmCHIL*-F (with *TDH3p*-R) | AGAACTTAGTTTCGAATAAACACACATAAACAAACAAAATGGCTACTGAAGAGGTTTTG |
| P167 | *GmCHIL*-R (with *CYC1t*-F) | CGTGACATAACTAATTACATGACTCGAGGTCGACGGTATCTCACTTGGACAACTCCTGC |
| P168 | *SmCHIL*-F (with *TDH3p*-R) | AACTTAGTTTCGAATAAACACACATAAACAAACAAAATGGAAATGGATCCAACCTTTG |
| P169 | *SmCHIL*-R (with *CYC1t*-F) | GTGACATAACTAATTACATGACTCGAGGTCGACGGTATCTCAACACTCCGTTGCAATAG |
| P170 | *OsCHIL*-F (with *TDH3p*-R) | AAGAACTTAGTTTCGAATAAACACACATAAACAAACAAAATGGGGACTGAGATCGCTAC |
| P171 | *OsCHIL*-R (with *CYC1t*-F) | GTGACATAACTAATTACATGACTCGAGGTCGACGGTATCTCACGCACTCAATAATGCCG |
| P172 | *AmUGT2^L11G^*-F (with *GAL10p*-R) | ATCCAAAAAAAAAGTAAGAATTTTTGAAAAACAAAATGAAAGACACTTTGGTTCT |
| P173 | *AmUGT2^L11G^*-R (with *FBA1t*-F) | TAAAAAACTATATCAATTAATTTGAATTAACCTATCTAAACAAGAATGGAGAATT |
| P174 | *AmUGT2^F196C^*-F (with *GAL10p*-R) | ATCCAAAAAAAAAGTAAGAATTTTTGAAAAACAAAATGAAAGACACTTTGGTTCT |
| P175 | *AmUGT2^F196C^*-R (with *FBA1t*-F) | TAAAAAACTATATCAATTAATTTGAATTAACCTATCTAAACAAGAATGGAGAATT |
| **Oligos for the construction of gRNA vectors** | | |
| P176 | pMEL10 backbone-F | GTTTTAGAGCTAGAAATAGCAAGTTAAAATAAGGCTAGTC |
| P177 | pMEL10 backbone-R | GATCATTTATCTTTCACTGCGGAGAAG |
| P178 | Seq-pMEL10-F | GCTGGCCTTTTGCTCACATG |
| P179 | Seq-pMEL10-R | CACCTTTCGAGAGGACGATG |
| P180 | *XI-1_gRNA* | GCAGTGAAAGATAAATGATCGCAATGCGATGTTAGTTTAGGTTTTAGAGCTAGAAATAGCAAG |
| P181 | *XII-1_gRNA* | GCAGTGAAAGATAAATGATCGGTATGTGCAGTTGATTCACGTTTTAGAGCTAGAAATAGCAAG |
| P182 | *XII-3_gRNA* | GCAGTGAAAGATAAATGATCCTTTATGCATAGAGCTAATTGTTTTAGAGCTAGAAATAGCAAG |
| **Flanking primers for PCR identification of DNA construct integration and gene deletion** | | |
| P183 | ID-*ELP3*-F | TGCCGCTTTCATTGTTTAATC |
| P184 | ID-*ELP3*-R | CAAACTAAAGAGCTTACACTTCG |
| P185 | ID-*FAS1p*-F | GTACATTGGGCCTTTTCATAC |
| P186 | ID-*FAS1p*-R | CTAGTTCAGCAGGTGTGGTAG |
| P187 | ID-*XII-1*-F | CTGGCAAGAGAACCACCAAT |
| P188 | ID-*XII-1*-R | GGACGACAACTACGGAGGAT |
| P189 | ID-*XI-1*-F | CTTAATGGGTAGTGCTTGACACG |
| P190 | ID-*XI-1*-R | GAAGACCCATGGTTCCAAGGA |
| P191 | ID-*XI-3*-F | GTGCTTGATTTGCGTCATTC |
| P192 | ID-*XI-3*-R | CACATTGAGCGAATGAAACG |
| P193 | ID-*XII-3*-F | TGGGCAGCCTTGAGTAAATC |
| P194 | ID-*XII-3*-R | TGGCCAATTGTTCAGTCAAG |
| P195 | ID-*XII-4*-F | GAACTGACGTCGAAGGCTCT |
| P196 | ID-*XII-4*-R | CGTGAAATCTCTTTGCGGTAG |
| **Primers for constructing mutants** | | |
| P197 | pUC19 backbone-F | GGATCCCCGGGTACC |
| P198 | pUC19 backbone-R | AAGCTTGGCGTAATCATGG |
| P199 | Seq-pUC19-F | AGTTAGCTCACTCATTAGGCAC |
| P200 | Seq-pUC19-R | GCAACTGTTGGGAAGGGC |
| P201 | *MET13*-F | CGGCCAGTGAATTCGAGCTCGGTACCCGGGGATCCTTATAGGCTTAGTAGGATGGAATG |
| P202 | *MET13*-R | AGGAAACAGCTATGACCATGATTACGCCAAGCTTATGAAGATCACAGAAAAATTAGAGC |
| P203 | *MET13^S443F^*-F | ATCACTATAAACTTTCAACCTCAAG |
| P204 | *MET13^S443F^*-R | CTTGAGGTTGAAAGTTTATAGTGAT |
| P205 | pET28a backbone-F | CATATGGCTGCCGCG |
| P206 | pET28a backbone-R | AAGCTTGCGGCCGC |
| P207 | Seq-pET28a-F | CCTCCTTTCAGCAAAAAACCC |
| P208 | Seq-pET28a-R | TAATACGACTCACTATAGGGGAATTG |
| P209 | *AmUGT2*-F | AGCAGCGGCCTGGTGCCGCGCGGCAGCCATATGATGAAAGACACTTTGGTTCTATATCC |
| P210 | *AmUGT2*-R | GTGGTGGTGCTCGAGTGCGGCCGCAAGCTTCTATCTAAACAAGAATGGAGAATTTGGTG |
| P211 | *AmUGT2^L11G^*-F | GTTCTATATCCAGCTGGTGGTAAAGGTCAT |
| P212 | *AmUGT2^L11G^*-R | ATGACCTTTACCACCAGCTGGATATAGAAC |
| P213 | *AmUGT2^H184D^*-F | TTGATATGCCAGAAGATACTAAAGACAGAG |
| P214 | *AmUGT2 ^H184D^*-R | CTCTGTCTTTAGTATCTTCTGGCATATCAA |
| P215 | *AmUGT2^H184S^*-F | TTGATATGCCAGAAAGCACTAAAGACAGAG |
| P216 | *AmUGT2 ^H184S^*-R | CTCTGTCTTTAGTGCTTTCTGGCATATCAA |
| P217 | *AmUGT2^F117C^*-F | CTTGGACTTCTGTAACTATTCTACTACTCA |
| P218 | *AmUGT2 ^F117C^*-R | TGAGTAGTAGAATAGTTACAGAAGTCCAAG |
| P219 | *AmUGT2^F117D^*-F | CTTGGACTTCGATAACTATTCTACTACTCA |
| P220 | *AmUGT2 ^F117D^*-R | TGAGTAGTAGAATAGTTATCGAAGTCCAAG |
| P221 | *AmUGT2^F117G^*-F | CTTGGACTTCGGTAACTATTCTACTACTCA |
| P222 | *AmUGT2 ^F117G^*-R | TGAGTAGTAGAATAGTTACCGAAGTCCAAG |
| P223 | *AmUGT2^F117P^*-F | CTTGGACTTCCCGAACTATTCTACTACTCA |
| P224 | *AmUGT2 ^F117P^*-R | TGAGTAGTAGAATAGTTCGGGAAGTCCAAG |
| P225 | *AmUGT2^F196C^*-F | ATCTTACAGAGTCTGTCTTGATATTGCTAC |
| P226 | *AmUGT2 ^F196C^*-R | GTAGCAATATCAAGACAGACTCTGTAAGAT |
| P227 | *AmUGT2^F196Y^*-F | ATCTTACAGAGTCTATCTTGATATTGCTAC |
| P228 | *AmUGT2 ^F196Y^*-R | GTAGCAATATCAAGATAGACTCTGTAAGAT |

**^a^ Short overlaps to the fragments in the parentheses were included in corresponding primers**
